# Fully volumetric body composition analysis for prognostic overall survival stratification in melanoma patients

**DOI:** 10.1186/s12967-025-06507-1

**Published:** 2025-05-12

**Authors:** Katarzyna Borys, Georg Lodde, Elisabeth Livingstone, Carsten Weishaupt, Christian Römer, Marc-David Künnemann, Anne Helfen, Lisa Zimmer, Wolfgang Galetzka, Johannes Haubold, Christoph M. Friedrich, Lale Umutlu, Walter Heindel, Dirk Schadendorf, René Hosch, Felix Nensa

**Affiliations:** 1 Institute for Artificial Intelligence in Medicine, University Hospital Essen, Girardetstraße 2, 245131 Essen, Germany; 2 Institute of Diagnostic and Interventional Radiology and Neuroradiology, University Hospital Essen, Essen, Germany; 3 Institute of Dermatology, University Hospital Essen, Essen, Germany; 4 Department of Dermatology, University Hospital Münster, Münster, Germany; 5 Clinic for Radiology, University Hospital Münster, Münster, Germany; 6 Institute of Medical Informatics, Biometry and Epidemiology, University Hospital Essen, University of Duisburg-Essen, Essen, Germany; 7 Department of Computer Science, University of Applied Sciences and Arts Dortmund, Dortmund, Germany

**Keywords:** Body composition, Melanoma, Computed tomography, Overall survival, Prognostication, Cancer, Biomarkers

## Abstract

**Background:**

Accurate assessment of expected survival in melanoma patients is crucial for treatment decisions. This study explores deep learning-based body composition analysis to predict overall survival (OS) using baseline Computed Tomography (CT) scans and identify fully volumetric, prognostic body composition features.

**Methods:**

A deep learning network segmented baseline abdomen and thorax CTs from a cohort of 495 patients. The Sarcopenia Index (SI), Myosteatosis Fat Index (MFI), and Visceral Fat Index (VFI) were derived and statistically assessed for prognosticating OS. External validation was performed with 428 patients.

**Results:**

SI was significantly associated with OS on both CT regions: abdomen (P ≤ 0.0001, HR: 0.36) and thorax (P ≤ 0.0001, HR: 0.27), with lower SI associated with worse survival. MFI was also associated with OS on abdomen (P ≤ 0.0001, HR: 1.16) and thorax CTs (P ≤ 0.0001, HR: 1.08), where higher MFI was linked to worse outcomes. Lastly, VFI was associated with OS on abdomen CTs (P ≤ 0.001, HR: 1.90), with higher VFI linked to poor outcomes. External validation replicated these results.

**Conclusions:**

SI, MFI, and VFI showed substantial potential as prognostic factors for OS in malignant melanoma patients. This approach leveraged existing CT scans without additional procedural or financial burdens, highlighting the seamless integration of DL-based body composition analysis into standard oncologic staging routines.

**Graphical Abstract:**

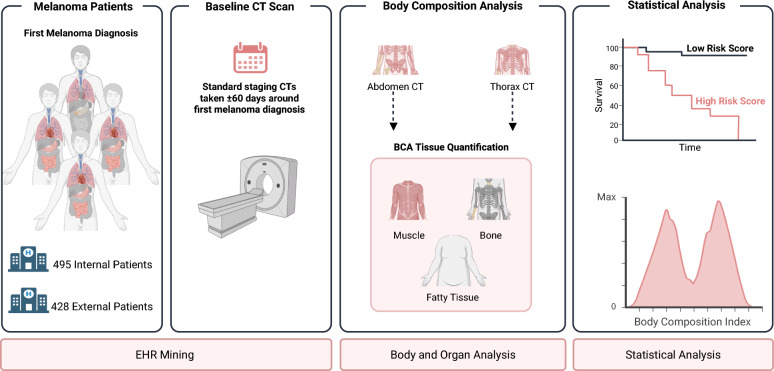

**Supplementary Information:**

The online version contains supplementary material available at 10.1186/s12967-025-06507-1.

## Introduction

Melanoma, known for its aggressive nature and early lymphogenous and hematogenous metastasis, presents substantial treatment challenges despite advances in systemic therapies such as immune checkpoint inhibition or targeted therapy with BRAF/MEK inhibitors [1, 2]. Prognostic stratification remains essential for optimizing individualized treatments and improving overall survival (OS). While prognostic factors like TNM staging, performance status (ECOG), metastasis location, and serum lactate dehydrogenase (LDH) levels are substantial, they often involve non-automated assessment.

Recent advancements in imaging and deep learning (DL) have leveraged body composition as a critical prognostic factor [3–6]. Computed Tomography (CT), routinely used in cancer staging, commonly contains unutilized physiological data, offering the potential for automatically evaluating patient health beyond primary tumor regions. In response, this study focuses on three body composition features derived from CT scans using DL: the Sarcopenia Index (SI), the Myosteatosis Fat Index (MFI), and the Visceral Fat Index (VFI). Sarcopenia, the loss of skeletal muscle mass and function, and Myosteatosis, characterized by fat infiltration into muscle tissue, are associated with poorer outcomes [7–10]. Additionally, increased VFI, the visceral to subcutaneous fat ratio, correlates with decreased OS in metastatic melanoma [11].

Unlike previous studies relying on surrogates such as Body Mass Index [12] or 2-D measurements from standard reference regions like the L3 vertebra [13], which can be less accurate in estimating muscle volume or tissue ratios, this study utilized a DL-based neural network to segment tissues from baseline CT scans automatically, and evaluated the prognostic value of fully volumetric SI, MFI, and VFI for OS. Simultaneously, this work showcases the seamless integration of DL-based body composition analysis (BCA) into routinely performed staging procedures.

## Materials and methods

### Internal cohort definition

**Fig. 1:**
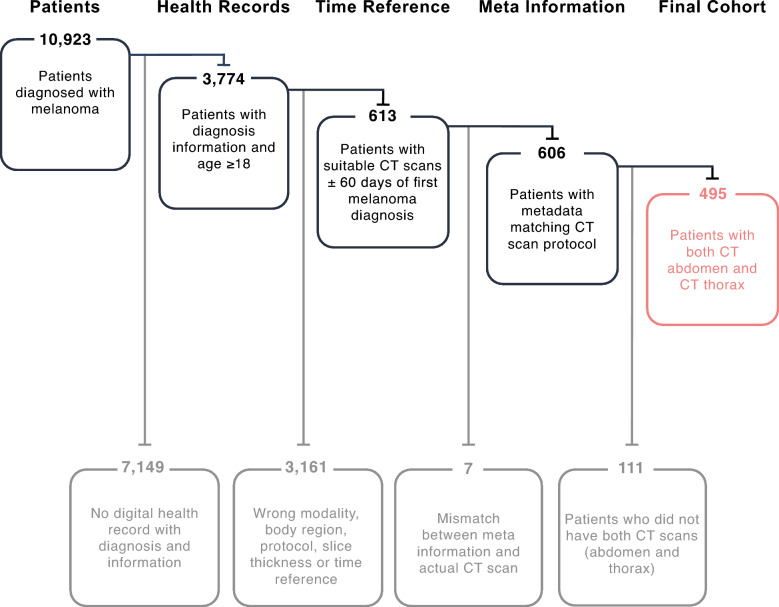
Schematic Acquisition Process for the Internal Cohort: Flowchart of the acquisition process, including image data filtering, clinical information Screening, time range adjustment, and meta-information filtering

Melanoma patients treated at the Department of Dermatology of the university hospital Essen, Germany, were identified using our internal server, leveraging the Fast Healthcare Interoperability Standard. Inclusion criteria required a CT scan of the abdomen, thorax, or whole body with a 5.0 mm slice thickness. Suitable whole-body CTs were cropped to the respective region of interest (abdomen or thorax), and scans with meta-data mismatches between the body regions detected by our segmentation tool and the series descriptions were removed. Preference was given to soft reconstruction kernels, while liver-only CTs were excluded due to limited region coverage. Patients lacking clinical information (diagnosis date, sex, age, and survival information) or younger than 18 were excluded. Further refinement included only those CTs taken within ± 60 days around the first melanoma diagnosis. Lastly, only patients having both CT regions (abdomen and thorax) were included for comparability between both regions, leading to a cohort of 495 eligible patients. The cohort acquisition process is shown in Fig. 1.

### External validation cohort definition

The external validation cohort comprised CT scans from melanoma patients treated at the Department of Dermatology of the university hospital Münster, Germany. The cohort’s inclusion criteria mirrored those of the internal cohort, requiring a baseline CT scan with the same slice thickness and kernels obtained within ± 60 of the first melanoma diagnosis. The only distinction was the external cohort's absence of thoracic CT scans. Ultimately, 428 patients were included for external validation of the statistical results derived from the internal cohort.

### Body composition analysis

Body composition was quantified using the Body and Organ Analysis (BOA) [14], which combines an nnU-Net neural network for BCA [15] with the TotalSegmentator [16], achieving a mean body voxel coverage of 93% ± 2%. Muscle, bone, intra- and intermuscular adipose tissue (IMAT), visceral adipose tissue (VAT), and total adipose tissue (TAT) were extracted using this tool. Additionally, the BOA enabled the identification of fully represented body regions (mediastinum, abdominal cavity, thoracic cavity), which were relevant for matching the scanned body site with CT metadata and identifying discrepancies.

SI was calculated as total muscle mass normalized to total bone mass, accounting for height differences across patients. This calculation assumes that bone volume remains relatively constant over time, compared to bone density, and can serve as a normalization parameter [17].

$$SI = \frac{Muscle\, [mL]}{Bone \,[mL]}$$

MFI was defined as the proportion of IMAT and TAT, measuring the fatty infiltration of muscular tissues as a percentage of the overall adipose tissue. It assesses the extent of myosteatosis, which is linked to cancer-related cachexia [18, 19].

$$MFI = \frac{Intra- and \,intermuscular\, adipose\, tissue \,[mL]}{Total \,Adipose\, Tissue\, [mL]}*100$$

VFI was defined as the VAT to SAT ratio, measuring the body’s total visceral fat proportion. This index is potentially prognostic because visceral fat is linked to greater endocrine and metabolic activity and has higher concentrations of inflammatory cells. Consequently, it can contribute to chronic low-grade systemic inflammation [20, 21].

$$VFI = \frac{Visceral \,Adipose\, Tissue\, [mL]}{Subcutaneous\, Adipose\, Tissue\, [mL]}$$

As visceral fat is mainly represented in the abdominal area, the VFI was only extracted from abdomen CT scans of the internal and external cohorts.

### Statistical analysis

Univariate and multivariate analyses were conducted, with OS as the primary endpoint. Survival time was measured in months from the first melanoma diagnosis until death from any cause or last contact for censoring, and median survival times were estimated using the Kaplan–Meier method. Analyses were performed separately for abdominal and thoracic CT scans to account for tissue distribution differences between both regions.

Python (version 3.10.12) and the packages lifelines 0.26.4 [22], shap [23], Scikit-Survival 0.17.2 [24], scipy 1.13.1 [25], optuna 2.10.1, [26], pandas 1.5.3 [27], and FHIR-PYrate 0.2.1 [28] were used.

Univariate Kaplan-Meier and Cox Regression analyses were applied to each CT region to estimate P-values, hazard ratios (HRs), and confidence intervals (CIs). Multivariate Cox regression was employed to evaluate the prognostic value of indices along with clinical baseline parameters (sex, M status, and age at diagnosis). Sex and M status were treated as dichotomous variables, while BCA parameters and age were continuous. Multivariate Cox regression models were fitted with and without each marker to assess their added value, and the Akaike Information Criterion (AIC) was calculated for each model.

Lastly, Gradient-boosted trees using Cox’s proportional Hazard loss function were trained to predict log HRs. First, for each model, the dataset was split into 70% for training and 30% for testing, ensuring stratification by the event status to maintain an equal distribution. The training set was then subjected to fivefold cross-validation, allowing each data point within the training set to serve as both training and validation data across different folds. Hyperparameter optimization was conducted via Optuna [26] to determine the best model configurations. Final predictions on the held-out test set were obtained by averaging the outputs from all models trained during cross-validation, forming an ensemble while maintaining consistent hyperparameters across models. Feature importance was assessed through permutation, with results averaged across all folds. Predictions were compared to the median hazard of the training data, and high-risk and low-risk patients were identified using the test cohort. Survival functions were then estimated using the Kaplan-Meier method. Finally, the models were evaluated on an external cohort.

Additionally, to assess the isolated impact of BCA indices on survival prediction, a baseline ML model using only age at diagnosis, sex, and M status was trained under the same conditions as the previous models. The Restricted Mean Survival Time (RMST) difference was then calculated between each BCA-enhanced model and the baseline model.

## Results

### Internal cohort definition

The internal cohort (N = 495, 43% female) had a median diagnosis age of 62 years [interquartile range (IQR) 51–74]. The median CT-to-diagnosis interval was 30 days [IQR 17- 42]. A list of used scanner types can be found in Supplementary Table 1. By 2024–08-01, 177 (36%) patients had died with a median follow-up time of 46 months [IQR 18–76]. For 354 patients, the M status was known: 310 (88%) had no distant metastases (M0), and 44 (12%) had at least one metastasis (M1). The M status was unknown for the remaining 141 (29%) patients (Mx). Baseline characteristics are detailed in Table 1.

**Table 1:** Baseline characteristics of the internal cohort: Baseline characteristics of the internal cohort, stratified by sex and M status

Total (median [IQR] age in years)	495 (62 [51–74])					
Sex	Female			Male		
Total (%)	213 (43)			282 (57)		
Patients’ age (median [IQR] in years)	60 [49–74]			64 [52–74]		
Survival time (median [IQR] in months)	116 [37–120]			89 [33–152]		
M Status	M0	M1	Mx	M0	M1	Mx
Total (%)	139 (65)	16 (8)	58 (27)	171 (61)	28 (10)	83 (17)
Deceased patients (%)	38 (18)	7 (3)	23 (11)	58 (21)	10 (4)	41 (23)
Age (median [IQR] in years)	62 [49–75]	67 [57–81]	58 [48–68]	65 [52–75]	68 [55–74]	62 [52–73]

The median and IQR were reported for continuous values. *M status* Distant metastatic status, *IQR* Interquartile range

### External cohort definition

In the external cohort (N = 428, 49% female), the median age at diagnosis was 63 [IQR 50–73], and 126 (29%) patients had died. The median follow-up time was 42 months [IQR 29–62], and the M status was available for all patients, with 349 (82%) having no distant metastases (M0) and 79 (18%) having at least one distant metastasis. Supplementary Table 2 presents the external cohort’s baseline statistics.

### Body composition analysis

The internal cohort’s abdomen CT scans revealed that SI was lower in M1 patients compared to M0 patients, reflecting physical deterioration associated with disease progression. The MFI and VFI were higher in M1 patients, indicating increased muscle fat infiltration or reduced adipose tissue due to weight loss. For detailed statistics, refer to Table 2 and Supplementary Tables 3 and 4 for the internal thorax and external cohort’s results.

**Table 2:** Statistical description of indices derived from the internal cohort’s abdominal CT scans: The SI, MFI, and VFI median and IQR are also stratified by sex and M status

	Overall					
SI (median ([IQR])	2.69 [2.36–3.02]					
MFI (median ([IQR])	8.98 [7.45–10.90]					
VFI (median [IQR])	0.50 [0.29–0.73]					
Sex	Female			Male		
SI (median [IQR])	2.57 [2.27–2.85]			2.79 [2.48–3.14]		
MFI (median ([IQR])	8.50 [6.92–10.62]			9.16 [7.81–11.02]		
VFI (median [IQR)]	0.28 [0.20–0.38]			0.68 [0.51–0.85]		
M Status	M0	M1	Mx	M0	M1	Mx
SI (median [IQR])	2.56 [2.25–2.90]	2.33 [2.24–2.74]	2.60 [2.32–2.82]	2.84 [2.47–3.20]	2.57 [2.21–3.04]	2.79 [2.54–3.06]
MFI (median ([IQR])	8.57 [6.82–10.68]	9.48 [7.98–12.12]	8.20 [7.15–10.04]	8.90 [7.62–10.89]	10.08 [8.55–11.93]	9.58 [8.31–10.98]
VFI (median [IQR])	0.26 [0.19–0.37]	0.32 [0.28–0.46]	0.29 [0.19–0.38]	0.68 [0.51–0.90]	0.68 [0.63–0.78]	0.69 [0.52–0.82]

*IQR* Interquartile range, *SI* Sarcopenia Index, *MFI* Myosteatosis Fat Index, *VFI* Visceral Fat Index, *M status* Distant metastatic status

In addition, a Mann–Whitney U test was used to assess whether there were significant deviations between the markers across the internal and external abdominal cohort. However, no significant differences could be identified for SI (P = 0.26), MFI (P = 0.76), and VFI (P = 0.1) (see Supplemental Table 4).

### Univariate analysis

#### Univariate Kaplan-Meier analysis for the internal and external CT scans

Kaplan-Meier analysis using a log-rank test on the internal abdomen CTs showed significantly prolonged OS for patients with SI above the median and MFI and VFI below the median. This was consistent for internal thorax CTs and the external cohort. Detailed results and sex-specific analyses can be found in Supplementary Figs. 5–8.

#### Univariate cox regression for internal and External CT scans

**Fig. 2:**
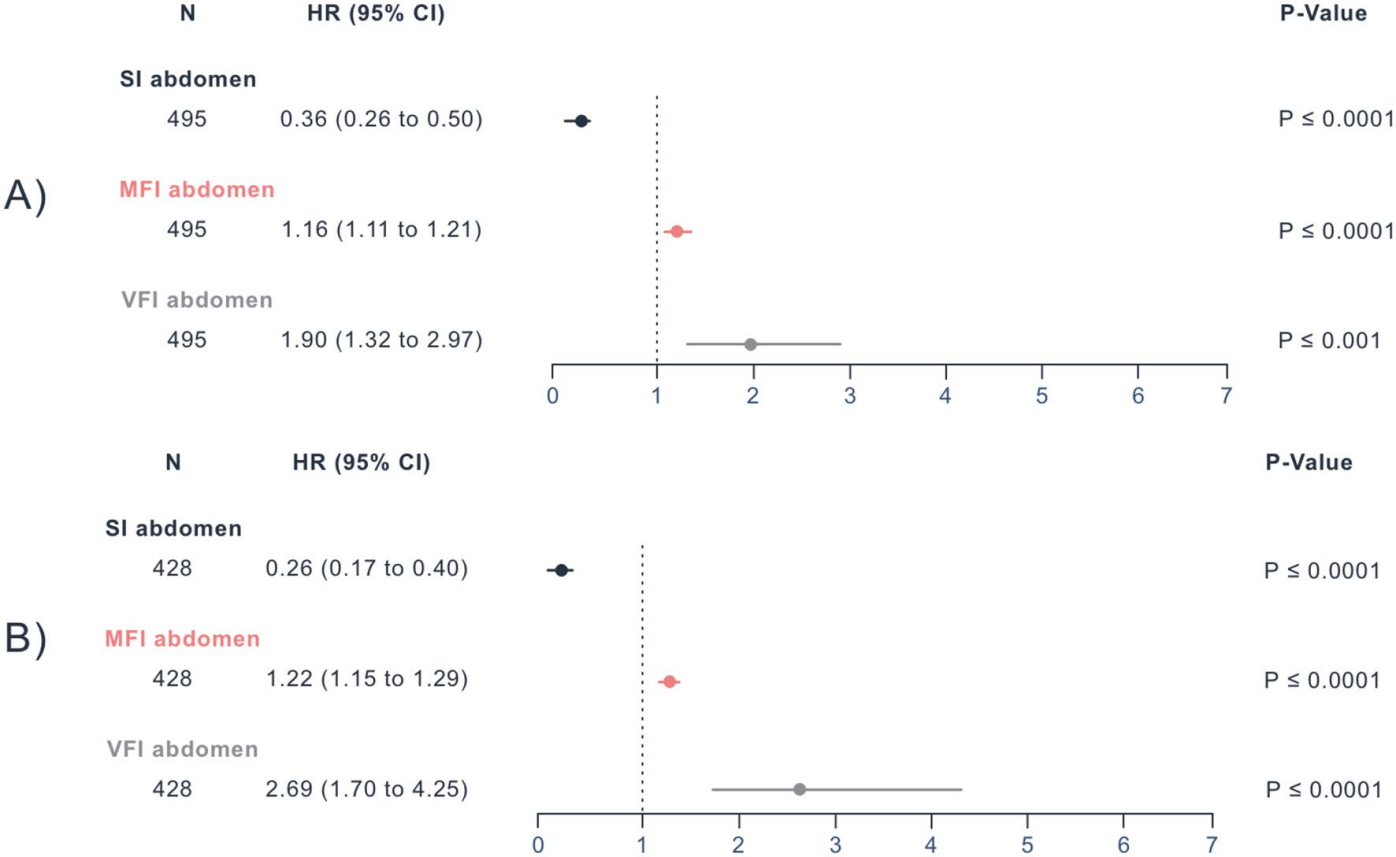
Univariate Cox regression for internal and external abdomen CT scans: Univariate Cox-Regression results obtained from the internal (**A**) and external (**B**) cohort’s abdominal CT scans showing P-Values, HRs, and 95% CIs for the SI, MFI, and VFI. *HR* Hazard Ratio, *CI* Confidence Interval, *SI* Sarcopenia Index, *MFI* Myosteatosis Fat Index, *VFI* Visceral Fat Index

For the internal abdomen CTs, SI (P ≤ 0.0001, HR: 0.36, 95% CI 0.26–0.50), MFI (P ≤ 0.0001, HR: 1.16, 95% CI 1.11–1.21), and VFI (P ≤ 0.001, HR: 1.90, 95% CI 1.32–2.97) were significantly associated with OS. These results were confirmed in external scans for SI (P ≤ 0.0001, HR: 0.26, 95% CI 0.17–0.40), MFI (P ≤ 0.0001, HR: 1.22, 95% CI 1.15–1.29), and VFI (P ≤ 0.0001, HR: 2.69, 95% CI 1.70–4.25) (Fig. 2). Results for the internal thorax scans can be found in Supplementary Fig. 9.

### Multivariate analysis

#### Multivariate cox regression for internal CT scans and external validation

**Fig. 3:**
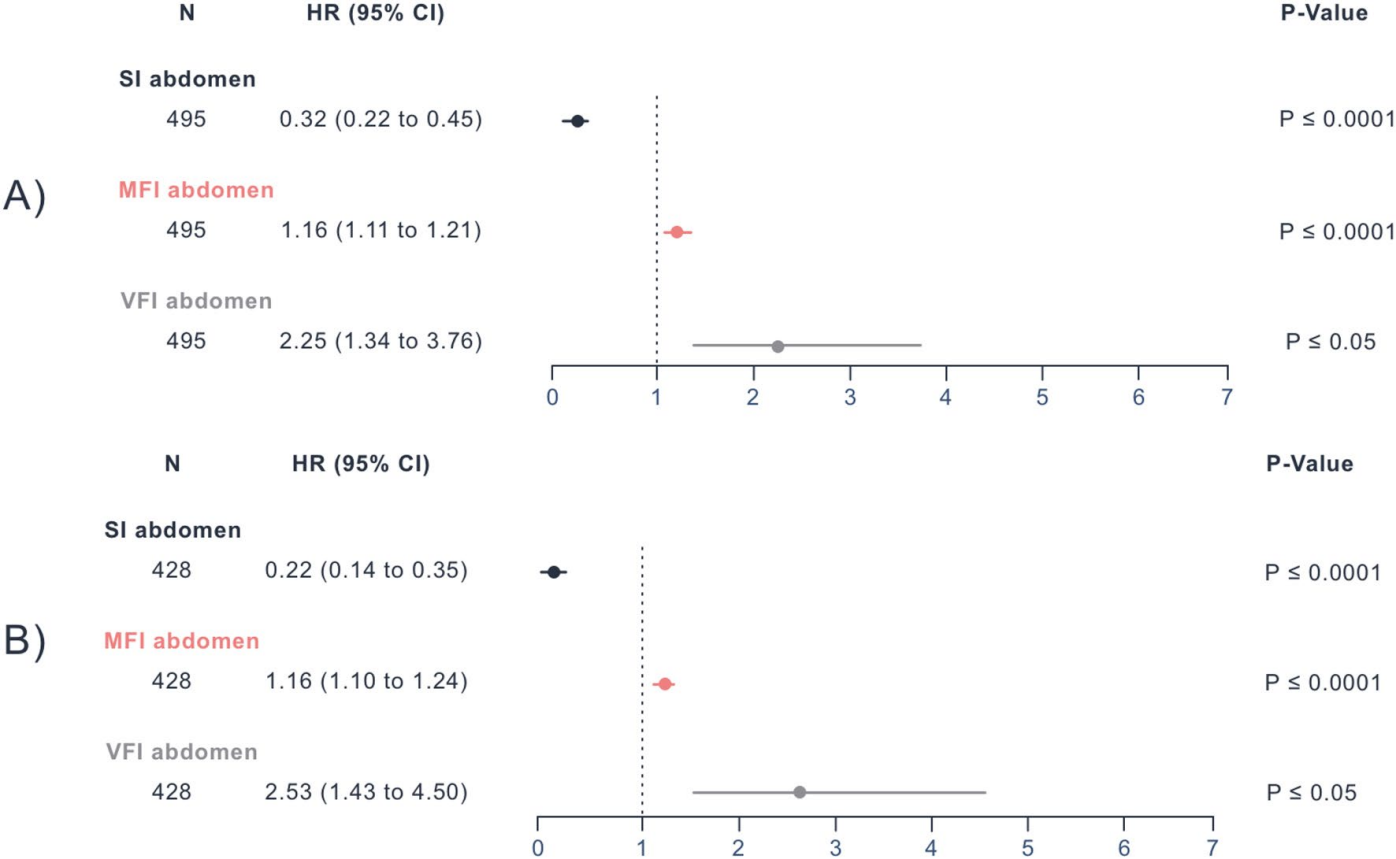
Multivariate Cox regression for internal and external abdomen CT scans: Multivariate Cox regression results obtained from the internal (**A**) and external (**B**) cohort’s abdominal CT scans showing P-Values, HRs, and 95% CIs for the SI, MFI, and VFI. *HR* Hazard Ratio, *CI* Confidence Interval, *SI* Sarcopenia Index, *MFI* Myosteatosis Fat Index, *VFI* Visceral Fat Index

In internal abdomen CTs, SI (P ≤ 0.0001, HR: 0.32, 95% CI 0.22–0.45), MFI (P ≤ 0.0001, HR: 1.16, 95% CI 1.11–1.21), and VFI (P ≤ 0.05, HR: 2.25, 95% CI 1.34–3.76) were significantly associated with OS in the presence of sex, age at diagnosis, and M status. These associations remained significant in the external cohort (Fig. 3) with SI (P ≤ 0.0001, HR: 0.22, 95% CI 0.14–0.35), MFI (P ≤ 0.0001, HR: 1.16, 95% CI 1.10–1.24), and VFI (P ≤ 0.05, HR: 2.53, 95% CI 1.43–4.50) and were also observed in the internal thorax CTs (Supplementary Fig. 10). Supplementary Figs. 11 and 12 show AIC plots comparing the multivariate Cox regression with and without each marker. These analyses demonstrated that models using the SI and MFI had lower AIC values, indicating a better fit. Only the VFI model did not improve significantly.

#### Machine learning for the internal CT scans and external validation

**Fig. 4:**
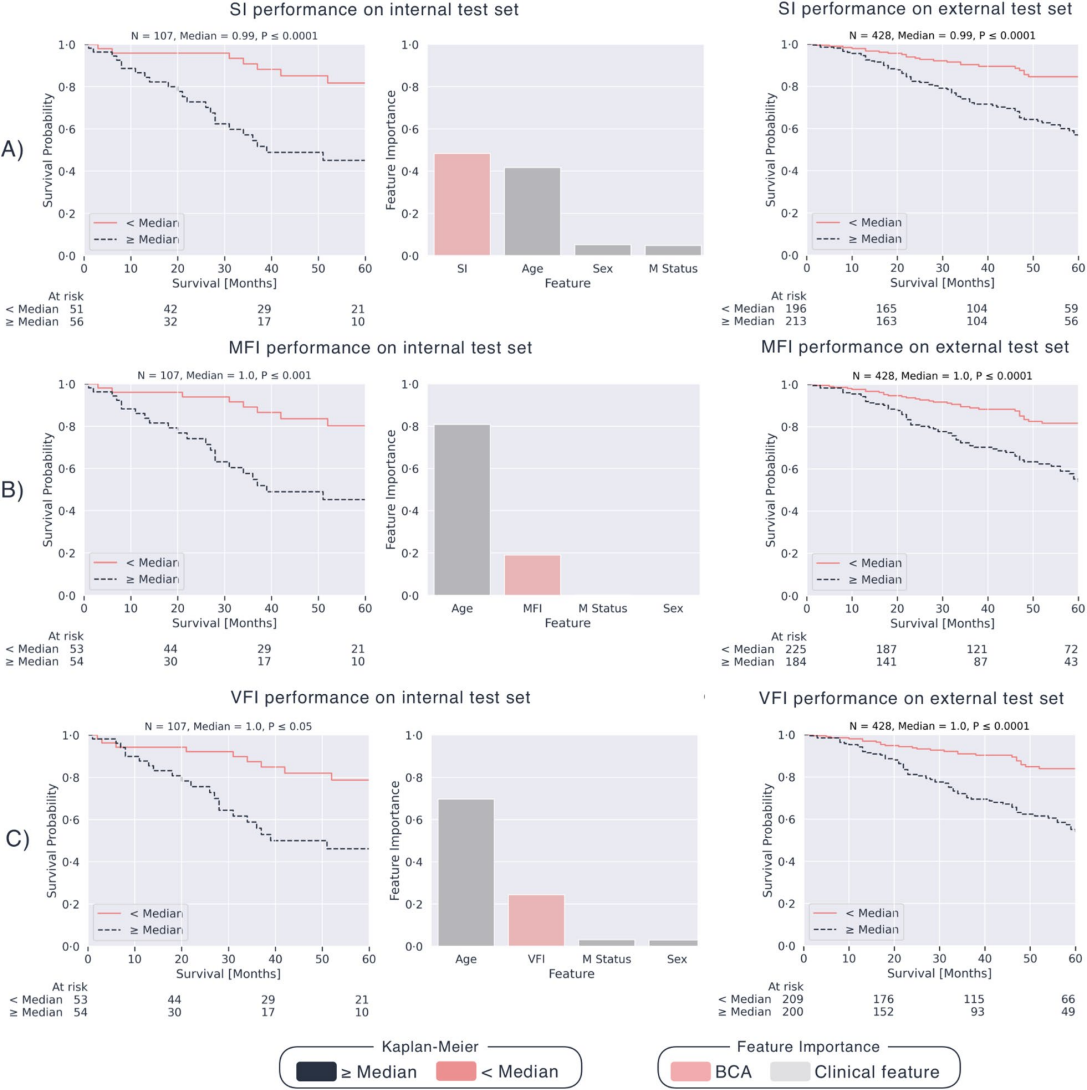
Multivariate machine learning results for internal and external abdomen CTs: Kaplan–Meier curves on the internal (left and middle column) and external (right column) abdomen CTs for the SI (**A**), MFI (**B**), and VFI (**C**) using the predicted risk scores of the multivariable abdomen-based BCA models trained and tested on patients with known M status (M0 + M1). Also, the averaged feature importance is shown for each model. *SI* Sarcopenia Index, *MFI* Myosteatosis Fat Index, *VFI* Visceral Fat Index, *BCA* Body Composition Analysis, *M status* Distant metastatic status

Models were trained on the internal abdomen and thorax CTs, including the features of sex, age at diagnosis, and M status. Importantly, only patients with known M status were included (M0 or M1). The resulting hyperparameters and Concordance indices are in Supplementary Table 13. Abdominal models effectively classified patients into low-risk and high-risk groups, with significant risk differentiation for SI (P ≤ 0.0001), MFI (P ≤ 0.001), and VFI (P ≤ 0.05) in the internal test set (N = 107). Feature importance analysis highlighted the indices’ substantial role in risk prediction. An extensive SHAP (SHapley Additive exPlanations) [23] analysis can be found in Supplementary Figs. 14 and 15. External validation confirmed the prognostic value of SI (P ≤ 0.0001), MFI (P ≤ 0.0001), and VFI (P ≤ 0.0001) (Fig. 4). Similar results were observed for internal thorax CT models (Supplementary Fig. 16).

**Fig. 5:**
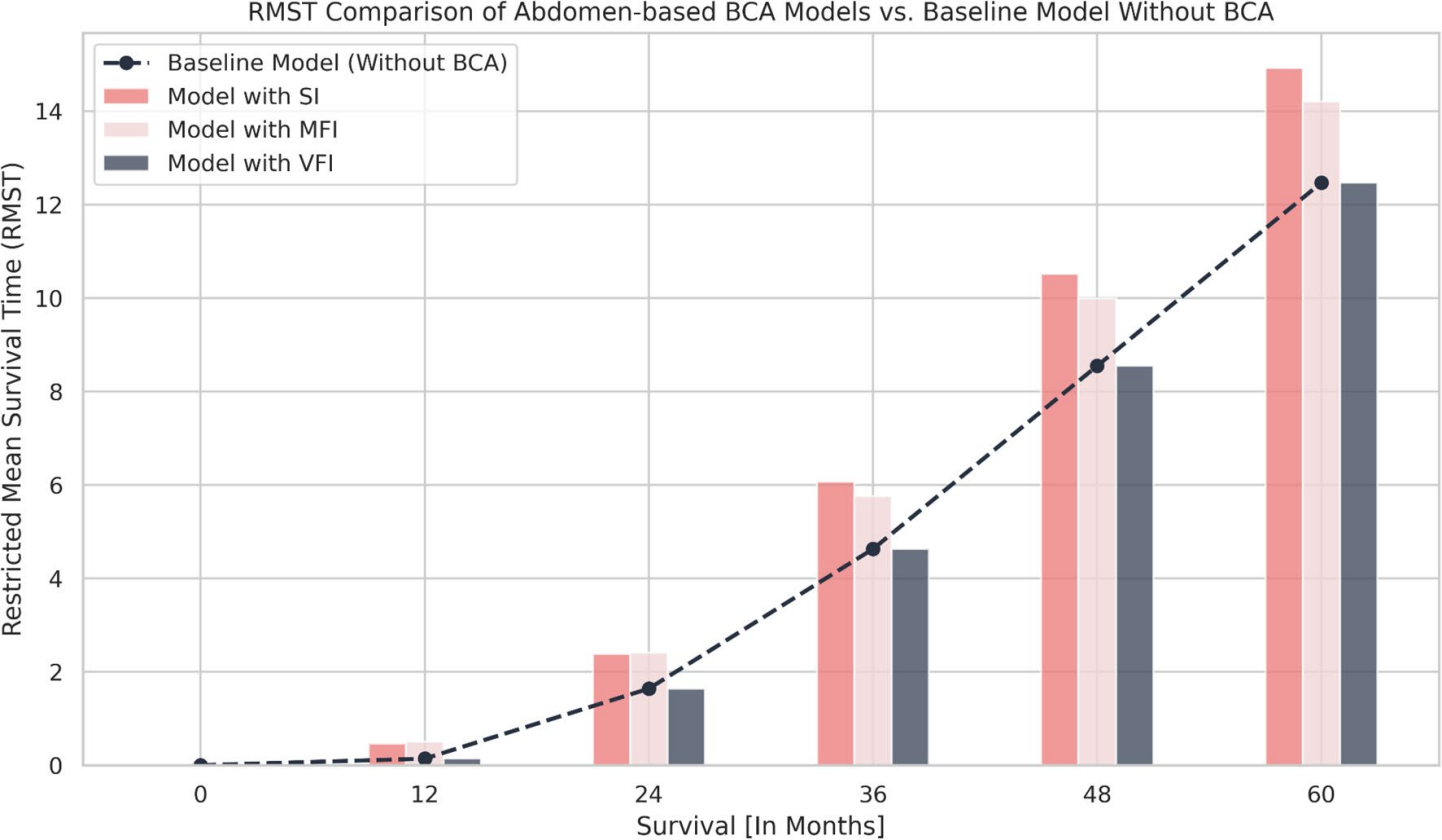
Restricted mean survival time differences between abdomen-based BCA models and the baseline model: The baseline ML model included only age at diagnosis, sex, and M status (M0 + M1), while the other models incorporated a respective BCA index in addition to these features. The RMST differences between models are presented over a 0 to 60-month range. *BCA* Body Composition Analysis, *ML* Machine Learning, *SI* Sarcopenia Index, *MFI* Myosteatosis Fat Index, *VFI* Visceral Fat Index, *RMST* Restricted Mean Survival Time

In addition, the RMST differences indicated that models incorporating abdomen-based SI and MFI were most effective in distinguishing high-risk from low-risk patients (Fig. 5).

While thorax-based SI and MFI also improved risk stratification compared to the baseline model, their performance was inferior to that of their abdominal counterparts. In contrast, VFI showed no improvement over the baseline model (Supplementary Fig. 17).

## Discussion

This study underscored the prognostic value of fully volumetric body composition indices derived from DL-based analysis of baseline CT scans in melanoma patients. The SI, MFI, and VFI were significant predictors of OS and effectively stratified patients into risk groups. External validation confirmed their prognostic power.

The SI was inversely associated with mortality, suggesting that higher muscle mass relative to bone leads to prolonged survival times. This is consistent with prior research highlighting the importance of muscle mass in cancer prognosis, where muscle shrinkage is associated with poorer outcomes [29–31]. The MFI showed a significant relationship with OS. Increased MFI values, indicating higher muscle fat infiltration, were associated with worse OS outcomes. This aligns with previous studies linking myosteatosis to cancer cachexia and adverse outcomes [32, 33]. The relation between MFI and disease progression suggests that increased muscle fat infiltration could reflect systemic deterioration and metabolic dysregulation [34, 35]. Higher VFI values were associated with shorter OS, highlighting the role of visceral fat in reinforcing inflammation that can adversely affect prognosis [36–38]. The consistency of these results across both the internal and external validation cohort highlights the reliability of these indices as prognostic tools. Additionally, the robustness of the results across different CT scan regions (abdomen and thorax) reinforces their validity on the two most common CT protocols.

Integrating DL-based body composition analysis into routine oncologic care offers several clinical advantages. Utilizing standard CT scans acquired during staging does not require additional procedures, limiting patient burden and procedural costs. This also enables opportunistic screening for body composition in clinical routines beyond staging and could provide a valuable source of additional diagnostic information [39–41]. Moreover, the indices could enhance individualized treatment planning, leading to more informed decision-making and potentially improving patient management strategies in melanoma. Also, these indices could aid in identifying patients at higher risk of unfavorable outcomes, allowing for early physical and nutritional interventional strategies or closer monitoring of these individuals.

A limitation of our study is the use of the median values of each index as cut-off points for Kaplan–Meier analyses. Alternative thresholds might have presented different risk stratifications, potentially affecting the observed prognostic value. In response, we included spline regressions in Supplementary Fig. 18 to explore the non-linear relationship between each index and the hazard function, offering a more nuanced understanding of the prognostic impact across their range. Another limitation is the lack of stratification by M status during the primary statistical analyses, which could have provided further insights into the prognostic value of these indices in different disease stages. Given the predominance of M0 in our cohort, stratification was only feasible for this group. To highlight the potential for the early detection of high-risk patients, we conducted the multivariate ML analysis for M0 patients, revealing that SI and MFI were significant predictors of OS (Supplementary Figs. 19 and 20). Future studies should aim for balanced distributions, enabling an analysis across various stages. Lastly, this study did not incorporate additional clinical variables, such as genomic profiles, laboratory values, and treatment details. Including these factors could provide a more comprehensive understanding of BCA in the context of melanoma outcomes. Future studies should integrate such variables and balanced distributions across different stages to enable analyses that account for the complexity of disease presentation and progression. Further research should also explore the longitudinal BCA in melanoma patients, particularly in the context of systemic treatments. These future directions are essential to fully understand BCA’s role as a supplemental factor and to refine its potential as an automated approach in clinical oncology.

Our study highlights the significant prognostic value of DL-based body composition analysis in melanoma patients. The SI, MFI, and VFI provide valuable information that can contribute to the prognostic landscape for melanoma, enabling more tailored and personalized treatment strategies without additional financial or procedural burdens.

## Supplementary Information

Supplementary Material 1.

## Data Availability

The dataset used in this study is not publicly available. Individuals or academic organizations interested in utilizing this dataset must submit a detailed request to [Data-Governance@uk-essen.de], which will be reviewed on a case-by-case basis.

## Abbreviations

BCA: Body Composition Analysis
CI: Confidence Interval
CT: Computed Tomography
DL: Deep Learning
HR: Hazard Ratio
ML: Machine Learning
MFI: Myosteatosis Fat Index
OS: Overall Survival
RMST: Restricted Mean Survival Time
SI: Sarcopenia Index
VFI: Visceral Fat Index
