# Correction: Fully volumetric body composition analysis for prognostic overall survival stratification in melanoma patients

**DOI:** 10.1186/s12967-025-06633-w

**Published:** 2025-05-28

**Authors:** Katarzyna Borys, Georg Lodde, Elisabeth Livingstone, Carsten Weishaupt, Christian Römer, Marc-David Künnemann, Anne Helfen, Lisa Zimmer, Wolfgang Galetzka, Johannes Haubold, Christoph M. Friedrich, Lale Umutlu, Walter Heindel, Dirk Schadendorf, René Hosch, Felix Nensa

**Affiliations:** 1 Institute for Artificial Intelligence in Medicine, University Hospital Essen, Girardetstraße 2, 245131 Essen, Germany; 2 Institute of Diagnostic and Interventional Radiology and Neuroradiology, University Hospital Essen, Essen, Germany; 3 Institute of Dermatology, University Hospital Essen, Essen, Germany; 4 Department of Dermatology, University Hospital Münster, Münster, Germany; 5 Clinic for Radiology, University Hospital Münster, Münster, Germany; 6 Institute of Medical Informatics, Biometry and Epidemiology, University Hospital Essen, University of Duisburg-Essen, Essen, Germany; 7 Department of Computer Science, University of Applied Sciences and Arts Dortmund, Dortmund, Germany

**Correction: J Transl Med 23**,** 532 (2025)**

10.1186/s12967-025-06507-1

Following publication of the original article [1], the authors reported an error in the abstract’s result section.

It was published as:

SI was significantly associated with OS on both CT regions: abdomen (*P* ≤ 0.0001, HR: 0.36) and thorax (*P* ≤ 0.0001, HR: 0.27), with lower SI associated with prolonged survival.

It has now been corrected to:

SI was significantly associated with OS on both CT regions: abdomen (*P* ≤ 0.0001, HR: 0.36) and thorax (*P* ≤ 0.0001, HR: 0.27), with lower SI associated with worse survival.

The original article [1] has been updated.
