# Supplementary material for: Fully volumetric body composition analysis for prognostic overall survival stratification in melanoma patients
Source: J Transl Med. 2025 May 12;23:532. doi: 10.1186/s12967-025-06507-1 (PMC12067685; doi:10.1186/s12967-025-06507-1)
Supplement: Supplementary file 1 — Supplementary Material 1. [file 12967_2025_6507_MOESM1_ESM.docx]

Fully Volumetric Body Composition Analysis for Prognostic Overall Survival Stratification in Malignant Melanoma Patients

Supplementary Material

[Table 1: Description of Scanner Types in the Internal Cohort 2](#_w8gtxqre60v2)

[Table 2: Baseline Characteristics of the External Cohort 3](#_nwou2637ww2m)

[Table 3: Statistics of Indices Derived from the Internal Cohort’s Thoracic CT Scans 3](#_qlxe7spta9x4)

[Table 4: Statistics of Indices Derived from the External Cohort’s Abdomen CT Scans 4](#_ih8skdxv5b5j)

[Figure 5: Univariate Kaplan-Meier Analysis of the Sarcopenia Index on Internal and External Abdomen CT Scans 5](#_c2rfz4a5mkdf)

[Figure 6: Univariate Kaplan-Meier Analysis of the Myosteatosis Fat Index on Internal and External Abdomen CT Scans 6](#_fe4sbv1vugfo)

[Figure 7: Univariate Kaplan-Meier Analysis of the Visceral Fat Index on Internal and External Abdomen CT Scans 7](#_60jeeh3w4pxo)

[Figure 8: Univariate Kaplan-Meier Analysis of the Sarcopenia Index and Myosteatosis Fat Index on Internal Thorax CT Scans 8](#_gpudd65szosy)

[Figure 9: Univariate Cox Regression of the Sarcopenia Index and Myosteatosis Fat Index on Internal Thorax CT Scans 8](#_93rsh9o90vx4)

[Figure 10: Multivariate Cox Regression of the Sarcopenia Index and Myosteatosis Fat Index on Internal Thorax CT Scans 9](#_fzwhdkoocbwh)

[Figure 11: Comparison of Multivariate Cox Regression With and Without Body Composition Indices for the Internal Abdomen CT models 9](#_msjznv6gx6hy)

[Figure 12: Comparison of Multivariate Cox Regression With and Without Body Composition Indices for the Internal Thorax CT Models 10](#_sw0gvtb66ndm)

[Table 13: Hyperparameters for GradientBoostedSurvival Models 10](#_vmaqe4258p0a)

[Figure 14: SHapley Additive exPlanations Analysis of Sarcopenia Index, Myosteatosis Fat Index, and Visceral Fat Index Models Using Internal Abdomen CT Test Data 12](#_5801ba5j4c6m)

[Figure 15: SHapley Additive exPlanations Analysis of Sarcopenia Index and Myosteatosis Fat Index Models Using Internal Thorax CT Test Data 13](#_lo8ec7x2mu0z)

[Figure 16: Multivariate Machine Learning Results on Internal Thorax CT Scans 14](#_uosszhdm0wi)

[Figure 17: Restricted Mean Survival Time Differences Between Thorax-based BCA Models and the Baseline Model 15](#_8pkn0pubgrpl)

[Figure 18: Spline Regression for Sarcopenia Index, Myosteatotis Fat Index, and Visceral Fat Index 16](#_gfofb6jxefj)

[Figure 19: Multivariate Machine Learning Results for Abdomen CT Scans in Patients With No Distant Metastases 17](#_te2jfbcb1qx)

[Figure 20: Multivariate Machine Learning Results for Thorax CT Scans in Patients With No Distant Metastases 18](#_b2lp0i3vc1qe)

# Table 1: Description of Scanner Types in the Internal Cohort

| **Manufacturer** | **Scanner Model** |
| --- | --- |
| GE MEDICAL SYSTEMS | BrightSpeed  Discovery 600  Discovery ST  LightSpeed VCT  Optima CT520 Series  Optima CT540  Optima CT660  Revolution CT  Revolution EVO  Revolution HD |
| PNMS | MX 16 |
| Philips | Brilliance 16  Brilliance 40  Brilliance 6  Brilliance 64  Brilliance Big Bore  GeminiGXL 6  HERMES Workstation  IQon - Spectral CT  Incisive CT  iCT 256 |
| SIEMENS | Biograph 64  Biograph128  Biograph20  Biograph64  Biograph64_Vision 600  Definition  Emotion 16  Emotion 16 (2007)  Emotion 16 (2010)  Emotion Duo  SOMATOM Definition  SOMATOM Definition AS  SOMATOM Definition AS+  SOMATOM Definition Edge  SOMATOM Definition Flash  SOMATOM Edge Plus  SOMATOM Force  SOMATOM Scope  SOMATOM go.Top  SOMATOM go.Up  Sensation 16  Volume Zoom |
|  |  |
| TOSHIBA | Activion16  Aquilion  Aquilion ONE  Aquilion PRIME  Astelion |

**Table 1: Description of Scanner Types in the Internal Cohort:** Overview of scanner manufacturers and models used for Computed Tomography scans from the internal cohort.

| Total (median [IQR] age in years) | 428 (63 [50 - 73]) | | | |
| --- | --- | --- | --- | --- |
| Sex | Female | | Male | |
| Total (%) | 208 (49) | | 220 (51) | |
| Patients’ age (median [IQR] in years) | 61 [49 - 73] | | 63 [52 - 74] | |
| Survival time (median [IQR] in months) | 106 [59 - 149] | | 86 [44 - 164] | |
| M Status | M0 | M1 | M0 | M1 |
| Total (%) | 175 (84) | 33 (16) | 174 (79) | 46 (21) |
| Deceased patients (%) | 33 (19) | 20 (61) | 34 (20) | 39 (85) |
| Age  (median [IQR] in years) | 61 [48 - 73] | 64 [49-73] | 63 [52 - 73] | 66 [56 - 77] |

Table 2: Baseline Characteristics of the External Cohort

**Table 2: Baseline Characteristics of the External Cohort:** Baseline statistical description of the external cohort stratified by sex and M status. The median and IQR were reported for continuous values. M Status: Distant metastatic status, IQR: interquartile range

# Table 3: Statistics of Indices Derived from the Internal Cohort’s Thoracic CT Scans

|  | **Overall** | | | | | |
| --- | --- | --- | --- | --- | --- | --- |
| SI (median [IQR]) | 1.83 [1.58 - 2.14] | | | | | |
| MFI (median [IQR]) | 14.20 [11.68 - 17.34] | | | | | |
| **Sex** | **Female** | | | **Male** | | |
| SI (median [IQR]) | 1.71 [1.53 - 1.94] | | | 1.96 [1.65 - 2.24] | | |
| MFI (median [IQR]) | 12.57 [10.25 - 15.35] | | | 15.54 [13.25 - 18.55] | | |
| **M Status** | M0 | M1 | Mx | M0 | M1 | Mx |
| SI (median [IQR]) | 1.72 [1.54 - 1.94] | 1.51 [1.42 - 1.73] | 1.72 [1.59 - 1.92] | 1.99 [1.62 - 2.30] | 1.74 [1.57 - 2.02] | 2.00 [1.74 - 2.21] |
| MFI (median [IQR]) | 12.80 [10.04 - 15.53] | 14.90 [12.32 - 16.48] | 11.71 [10.18 - 14.47] | 15.22 [13.19 - 18.56] | 16.49 [14.24 - 20.88] | 16.00 [12.93 - 18.37] |

**Table 3: Statistics of Indices Derived from the Internal Cohort’s Thoracic CT Scans**: The median and IQR for the SI, MFI, and VFI are stratified by sex and M status. SI: Sarcopenia Index, MFI: Myosteatosis Fat Index, VFI: Visceral Fat Index, M Status: Distant metastatic status, IQR: interquartile range

# Table 4: Statistics of Indices Derived from the External Cohort’s Abdomen CT Scans

|  | **Overall** | | | |
| --- | --- | --- | --- | --- |
| SI (median [IQR]) | 2.64 [2.34 - 2.97] | | | |
| SI (P-value) | 0.26 | | | |
| MFI (median [IQR]) | 8.84 [7.64 - 10.71] | | | |
| MFI (P-value) | 0.76 | | | |
| VFI (median [IQR]) | 0.53 [0.31 - 0.83] | | | |
| VFI (P-value) | 0.10 | | | |
| **Sex** | **Female** | | **Male** | |
| SI (median [IQR]) | 2.53 [2.28 - 2.82] | | 2.78 [2.45 - 3.08] | |
| MFI (median [IQR]) | 8.35 [7.31 - 10.34] | | 9.30 [8.02 - 11.14] | |
| VFI (median [IQR]) | 0.31 [0.23 - 0.41] | | 0.79 [0.62 - 1.03] | |
| **M Status** | M0 | M1 | M0 | M1 |
| SI (median [IQR])) | 2.54 [2.26 - 2.82] | 2.50 [2.32 - 2.81] | 2.80 [2.51 - 3.14] | 2.64 [2.32 - 2.90] |
| MFI (median ([IQR])) | 8.35 [7.40 - 10.27] | 8.35 [7.11 - 10.74] | 9.27 [7.84 - 11.02] | 9.46 [8.46 - 11.78] |
| VFI (median [IQR]) | 0.31 [0.22 - 0.41] | 0.31 [0.24 - 0.37] | 0.78 [0.59 - 1.00] | 0.86 [0.63 - 1.14] |

**Table 4:** **Statistics of Indices Derived from the External Cohort’s Abdomen CT Scans**: The median and IQR for the SI, MFI, and VFI are stratified by sex and M status. A Mann-Whitney U test obtained the reported P-values to assess whether there were significant differences between the markers across the internal and external cohort. SI: Sarcopenia Index, MFI: Myosteatosis Fat Index, VFI: Visceral Fat Index, M Status: Distant metastatic status, IQR: interquartile range

# Figure 5: Univariate Kaplan-Meier Analysis of the Sarcopenia Index on Internal and External Abdomen CT Scans


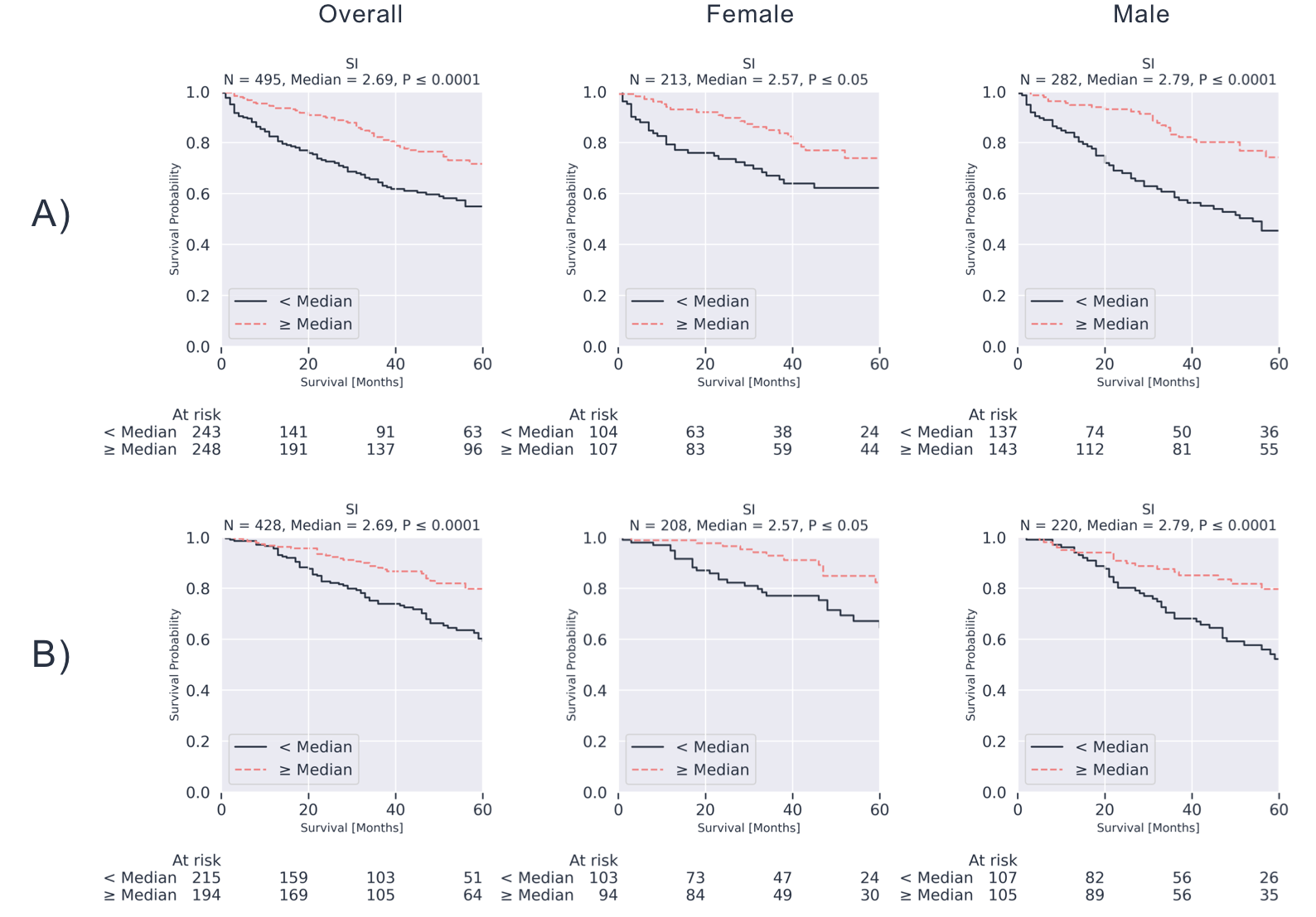

**Figure 5:** **Univariate Kaplan-Meier Analysis of the Sarcopenia Index on Internal and External Abdomen CT Scans:** Univariate Kaplan-Meier curves of the SI derived from the internal cohort’s (A) and external cohort’s (B) Abdomen CT scans. The SI was assessed in the overall cohort and distinct sex groups. SI: Sarcopenia Index

# Figure 6: Univariate Kaplan-Meier Analysis of the Myosteatosis Fat Index on Internal and External Abdomen CT Scans


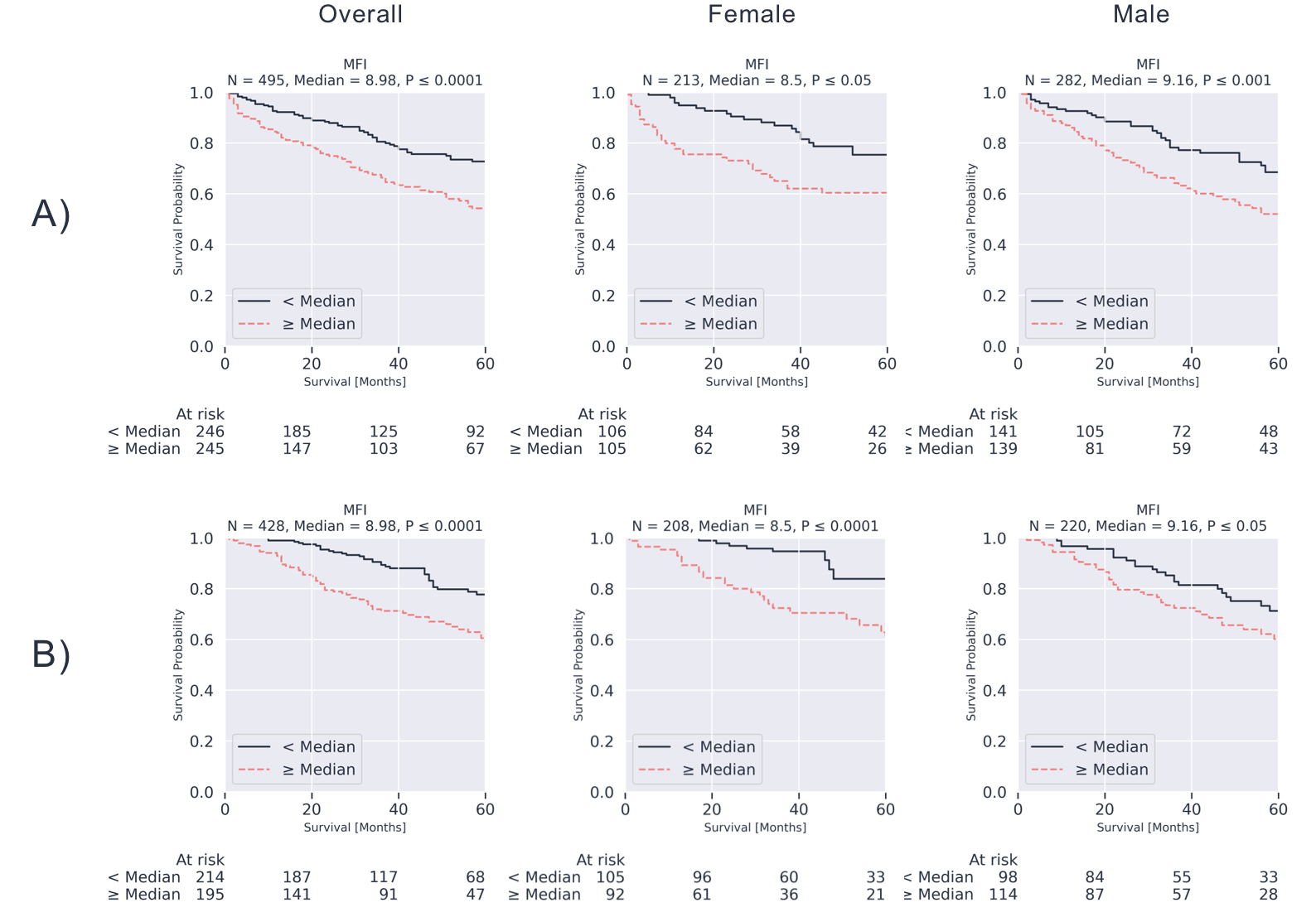

**Figure 6:** **Univariate Kaplan-Meier Analysis of the Myosteatosis Fat Index on Internal and External Abdomen CT Scans:** Univariate Kaplan-Meier curves of the MFI derived from the internal cohort’s (A) and external cohort’s (B) Abdomen CT scans. The MFI was assessed in the overall cohort and distinct sex groups. MFI: Myosteatosis Fat Index

# Figure 7: Univariate Kaplan-Meier Analysis of the Visceral Fat Index on Internal and External Abdomen CT Scans


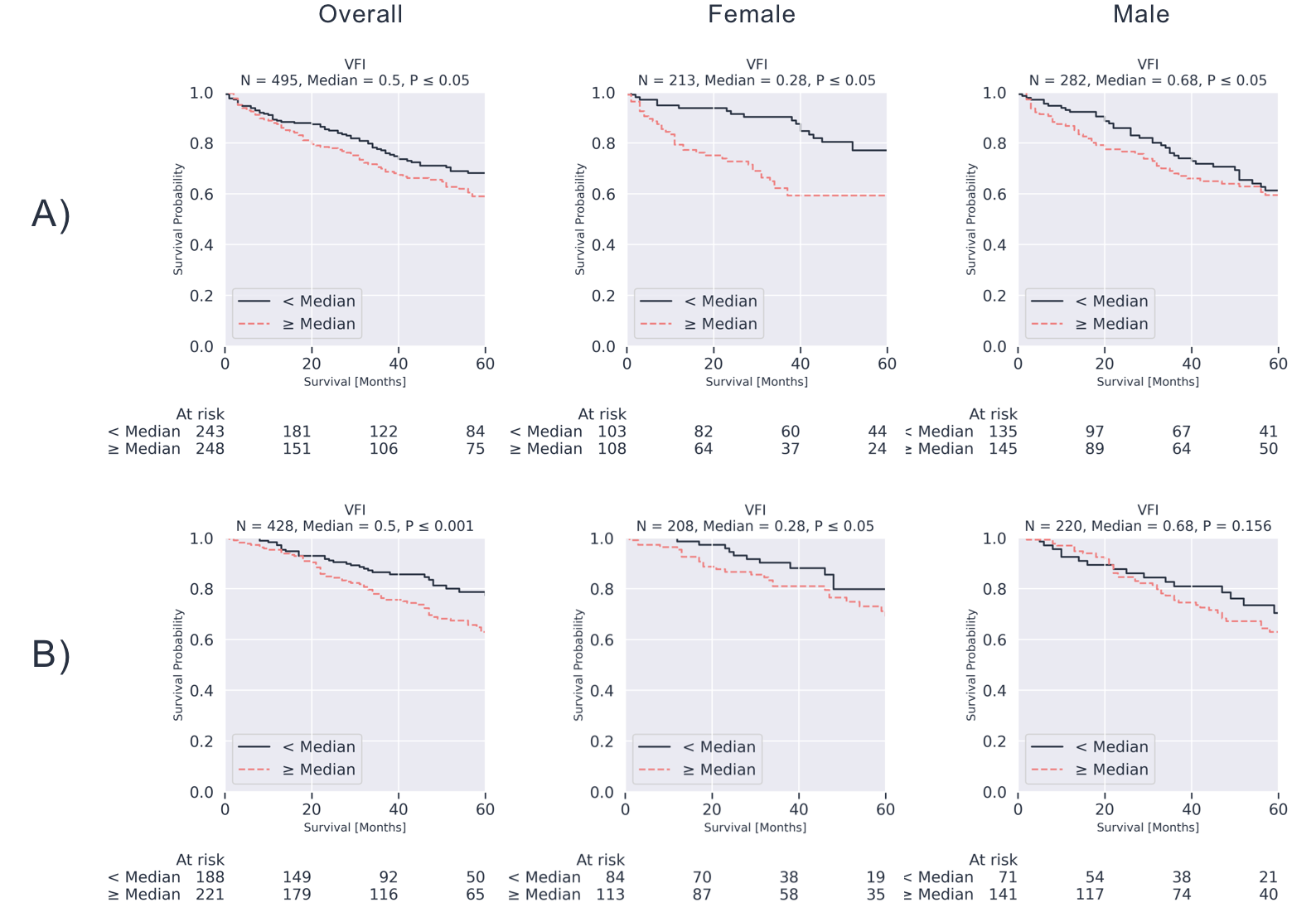

**Figure 7:** **Univariate Kaplan-Meier Analysis of the Visceral Fat Index on Internal and External Abdomen CT Scans:** Univariate Kaplan-Meier curves of the VFI derived from the internal cohort’s (A) and external cohort’s (B) Abdomen CT scans. The VFI was assessed in the overall cohort and distinct sex groups. VFI: Visceral Fat Index

# Figure 8: Univariate Kaplan-Meier Analysis of the Sarcopenia Index and Myosteatosis Fat Index on Internal Thorax CT Scans


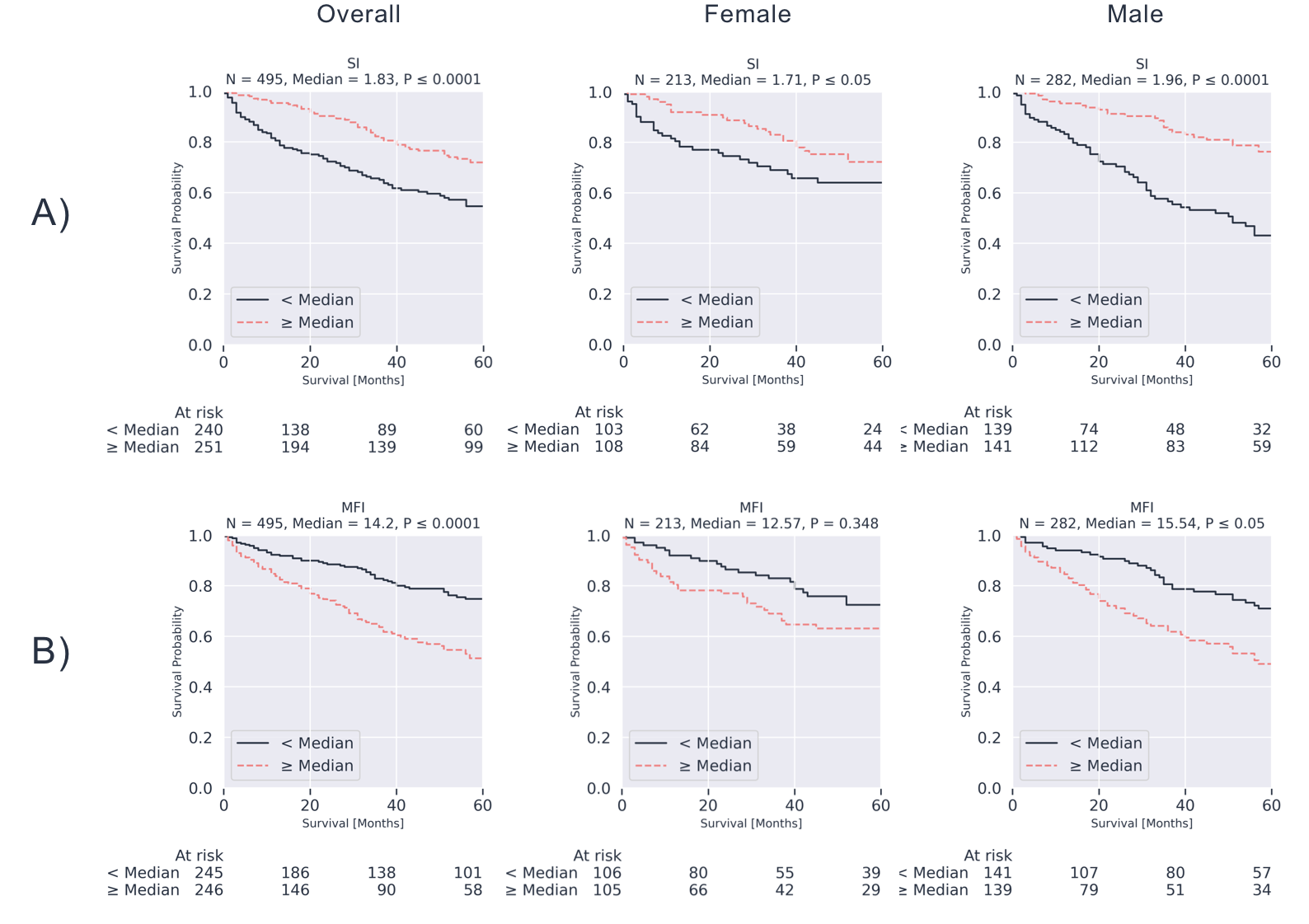

**Figure 8:** **Univariate Kaplan-Meier Analysis of the Sarrcopenia Index and Myosteatosis Fat Index on Internal Thorax CT Scans:** Univariate Kaplan-Meier curves of the SI and MFI derived from the internal cohort’s thorax CT scans. The SI (A) and MFI (B) were assessed in the overall cohort and distinct sex groups. SI: Sarcopenia Index, MFI: Myosteatosis Fat Index

# Figure 9: Univariate Cox Regression of the Sarcopenia Index and Myosteatosis Fat Index on Internal Thorax CT Scans


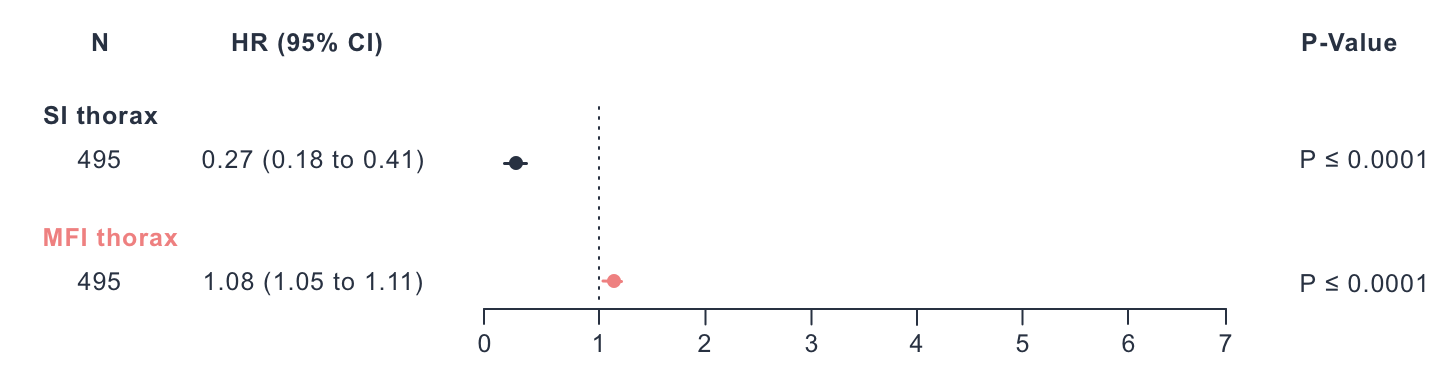

**Figure 9:** **Univariate Cox Regression of the Sarcopenia Index and Myosteatosis Fat Index on Internal Thorax CT Scans:** Univariate Cox Regression results of the SI and MFI derived from the internal cohort’s thorax CT scans showing P-Values, HRs, and 95% CIs. SI: Sarcopenia Index, MFI: Myosteatosis Fat Index, HR: Hazard Ratio, CI: Confidence Interval

# Figure 10: Multivariate Cox Regression of the Sarcopenia Index and Myosteatosis Fat Index on Internal Thorax CT Scans


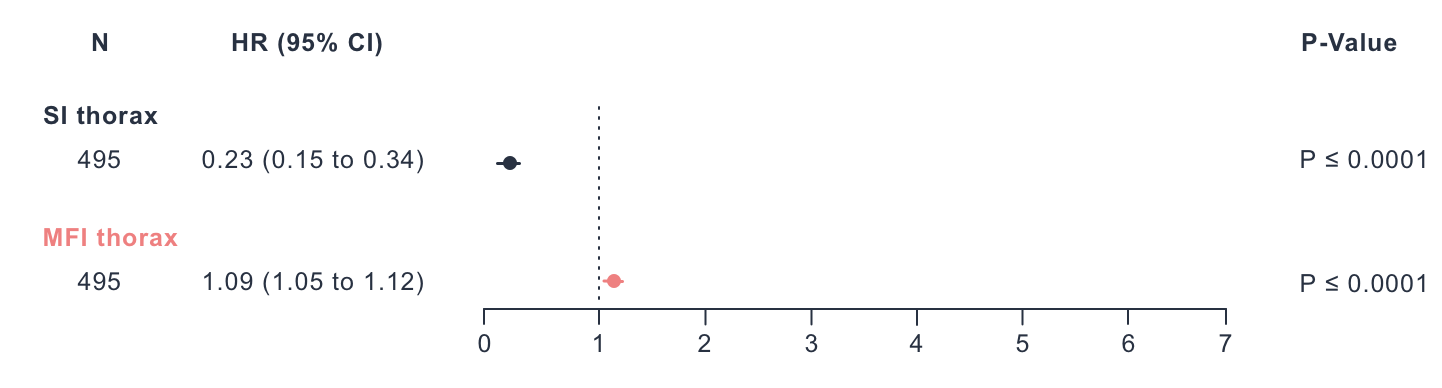

**Figure 10:** **Multivariate Cox Regression of the Sarcopenia Index and Myosteatosis Fat Index on Internal Thorax CT Scans:** Multivariate Cox Regression results of the SIand MFI derived from the internal cohort’s thorax CT scans showing P-Values, HRs, and 95% CIs. SI: Sarcopenia Index, MFI: Myosteatosis Fat Index, HR: Hazard Ratio, CI: Confidence Interval

# Figure 11: Comparison of Multivariate Cox Regression With and Without Body Composition Indices for the Internal Abdomen CT models


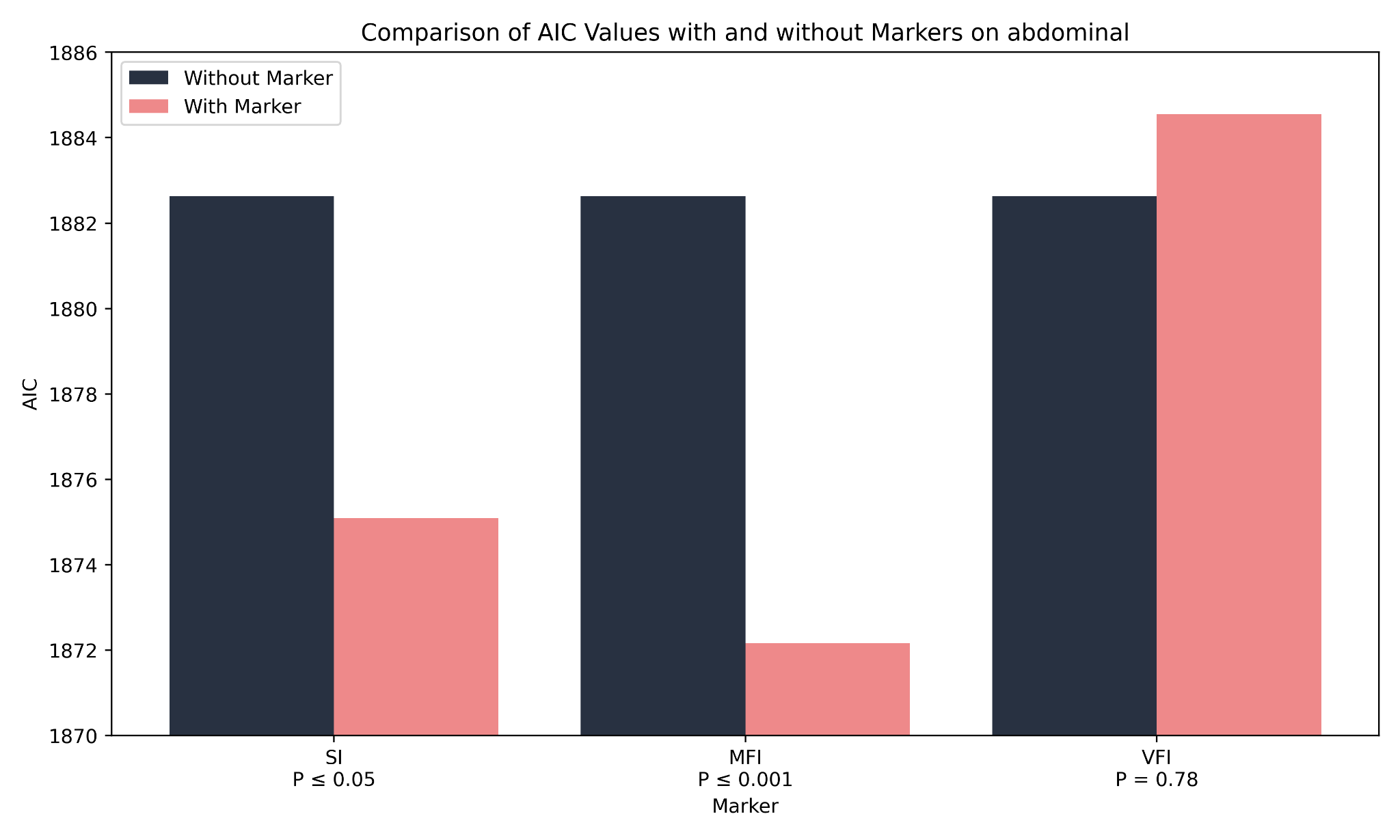

**Figure 11: Comparison of Multivariate Cox Regression With and Without Body Composition Indices for the Internal Abdomen CT Models:** This figure illustrates the AIC values for models trained on abdomen CT scans, comparing those with and without specific markers (SI, MFI, VFI). Bars represent the AIC values, with blue bars showing models without the respective marker and red bars showing models with the marker. P-values below the bars indicate the statistical significance of the difference between models with and without the marker, helping to assess whether including the marker significantly improved model fit. A lower AIC value suggests a better model fit relative to the number of parameters used. AIC: Akaike Information Criterion, SI: Sarcopenia Index, MFI: Myosteatosis Fat Index, VFI: Visceral Fat Index

# Figure 12: Comparison of Multivariate Cox Regression With and Without Body Composition Indices for the Internal Thorax CT Models


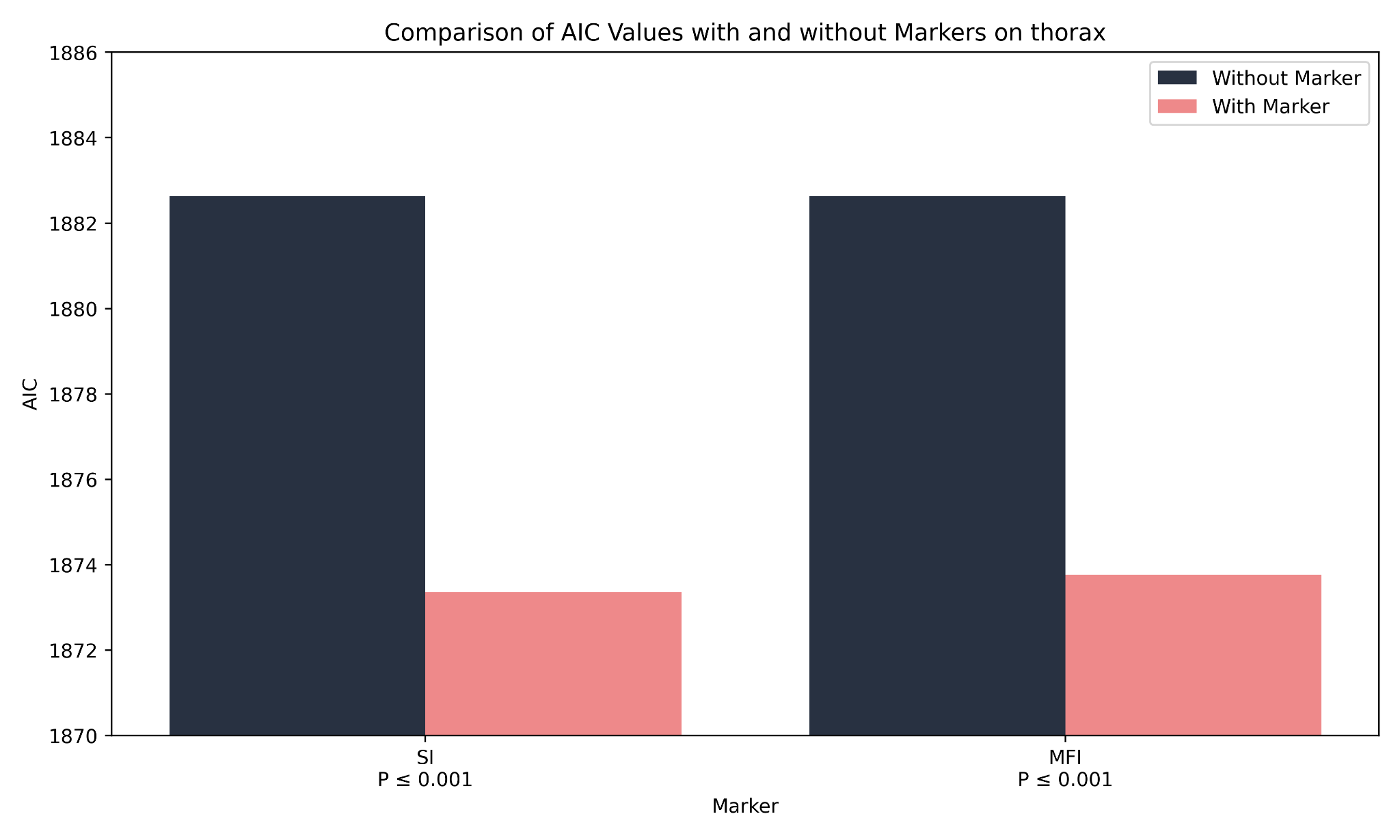

**Figure 12: Comparison of Multivariate Cox Regression With and Without Body Composition Indices for the Internal Thorax CT Models:** This figure illustrates the AIC values for models trained on thorax CT scans, comparing those with and without specific markers (SI, MFI, VFI). Bars represent the AIC values, with blue bars showing models without the respective marker and red bars showing models with the marker. P-values above the bars indicate the statistical significance of the difference between models with and without the marker, helping to assess whether including the marker significantly improved model fit. A lower AIC value suggests a better model fit relative to the number of parameters used. AIC: Akaike Information Criterion, SI: Sarcopenia Index, MFI: Myosteatosis Fat Index

# Table 13: Hyperparameters for GradientBoostedSurvival Models

| **Region** | **M Status Group** | **BCA Index** | **Learning Rate** | **Max Depth** | **Max Features** | **Subsample** | **Mean Concordance Index** |
| --- | --- | --- | --- | --- | --- | --- | --- |
| **Abdomen** | M01 | SI | 2.86e-03 | 4 | 0.20 | 0.70 | 0.67 ± 0.01 |
| **Thorax** | M01 | SI | 2.86e-03 | 4 | 0.20 | 0.70 | 0.69 ± 0.01 |
| **Abdomen** | M01 | MFI | 2.70e-04 | 1 | 0.75 | 0.80 | 0.71 ± 0.01 |
| **Thorax** | M01 | MFI | 2.70e-04 | 1 | 0.75 | 0.80 | 0.65 ± 0.02 |
| **Abdomen** | M01 | VFI | 1.06e-05 | 2 | 0.65 | 0.90 | 0.68 ± 0.02 |
| **Thorax** | M01 | VFI | - | - | - | - | - |
| **Abdomen** | M0 | SI | 2.61e-05 | 2 | 0.20 | 0.75 | 0.72 ± 0.05 |
| **Thorax** | M0 | SI | 2.61e-05 | 2 | 0.20 | 0.75 | 0.69 ± 0.07 |
| **Abdomen** | M0 | MFI | 2.70e-04 | 1 | 0.75 | 0.80 | 0.71 ± 0.03 |
| **Thorax** | M0 | MFI | 2.70e-04 | 1 | 0.75 | 0.80 | 0.64 ± 0.06 |
| **Abdomen** | M0 | VFI | 2.09e-04 | 1 | 0.70 | 0.75 | 0.69 ± 0.06 |
| **Thorax** | M0 | VFI | - | - | - | - | - |

**Table 13: Hyperparameters for GradientBoostedSurvival Models**: Hyperparameters used for each ensemble of GradientBoostingSurvival models, which were trained for each combination of region (abdomen and thorax) and index (SI, MFI, and VFI) separately. SI: Sarcopenia Index, MFI: Myosteatosis Fat Index, VFI: Visceral Fat Index

# Figure 14: SHapley Additive exPlanations Analysis of Sarcopenia Index, Myosteatosis Fat Index, and Visceral Fat Index Models Using Internal Abdomen CT Test Data


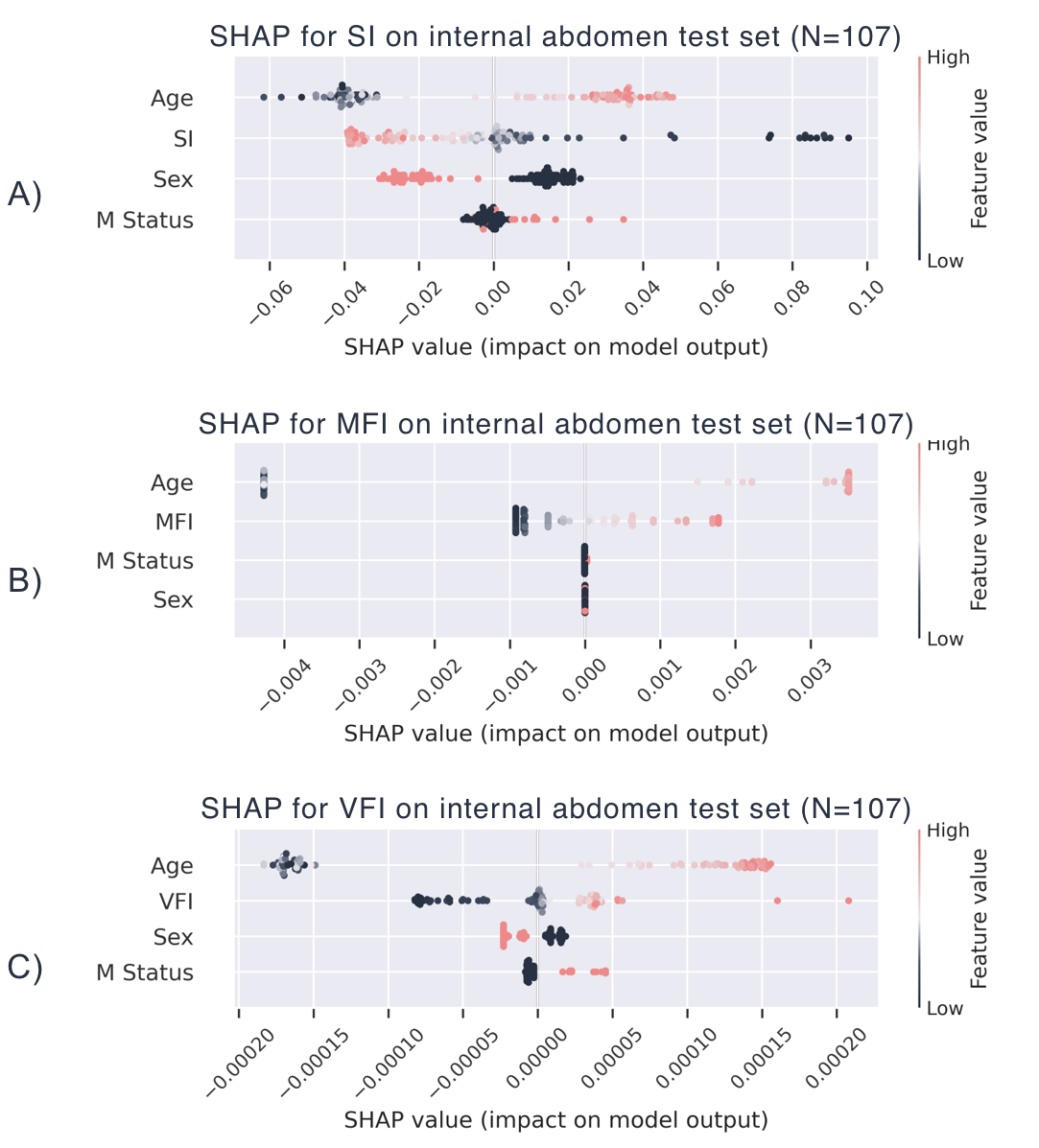

**Figure 14:** **SHapley Additive exPlanations Analysis of Sarcopenia Index, Myosteatosis Fat Index, and Visceral Fat Index Models Using Internal Abdomen CT Test Data:** SHAP provides insights into how each feature affects the model's prediction (risk score). These analyses show whether individual feature values (high or low) increase or decrease the predicted risk scores for patients. SHAP plots are presented for SI (A), MFI (B), and VFI (C). SI: Sarcopenia Index, MFI: Myosteatosis Fat Index, VFI: Visceral Fat Index, SHAP: SHapley Additive exPlanations

# Figure 15: SHapley Additive exPlanations Analysis of Sarcopenia Index and Myosteatosis Fat Index Models Using Internal Thorax CT Test Data


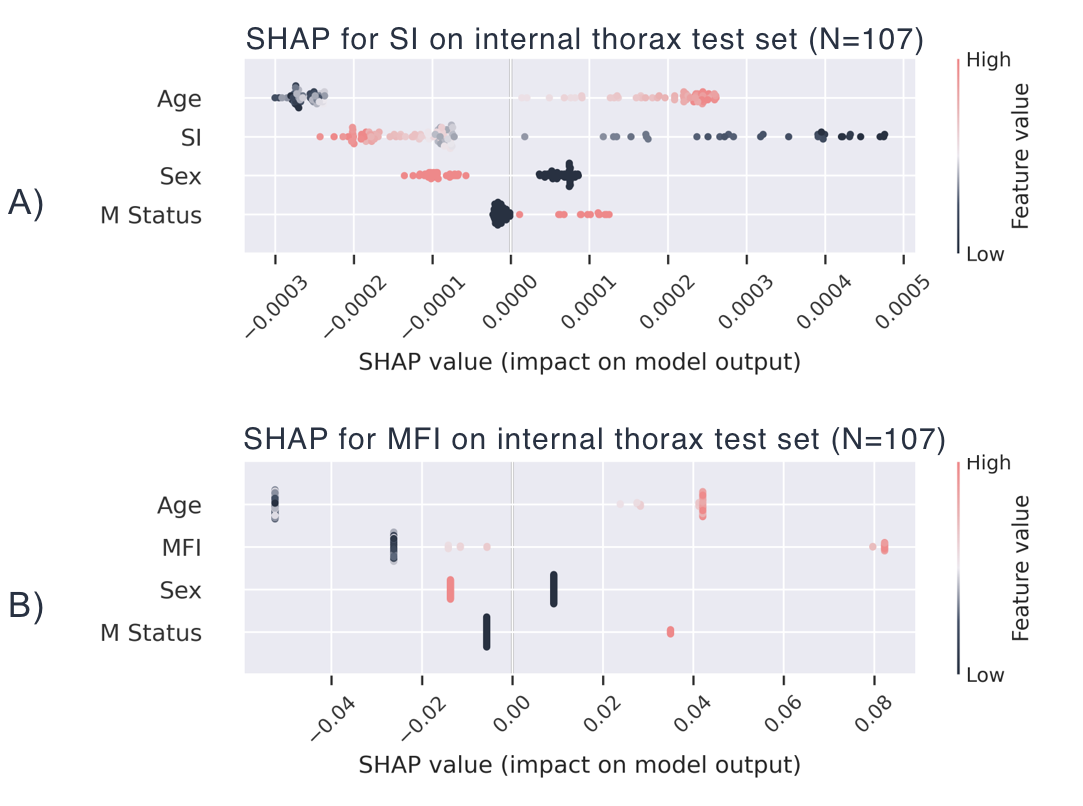

**Figure 15:** **SHapley Additive exPlanations Analysis of Sarcopenia Index and Myosteatosis Fat Index Models Using Internal Thorax CT Test Data**: SHAP analysis of models trained on internal thorax CTs for SI (A) and MFI (B). The SHAP results were obtained from the test dataset. SI: Sarcopenia Index, MFI: Myosteatosis Fat Index, VFI: Visceral Fat Index, SHAP: SHapley Additive exPlanations

# Figure 16: Multivariate Machine Learning Results on Internal Thorax CT Scans


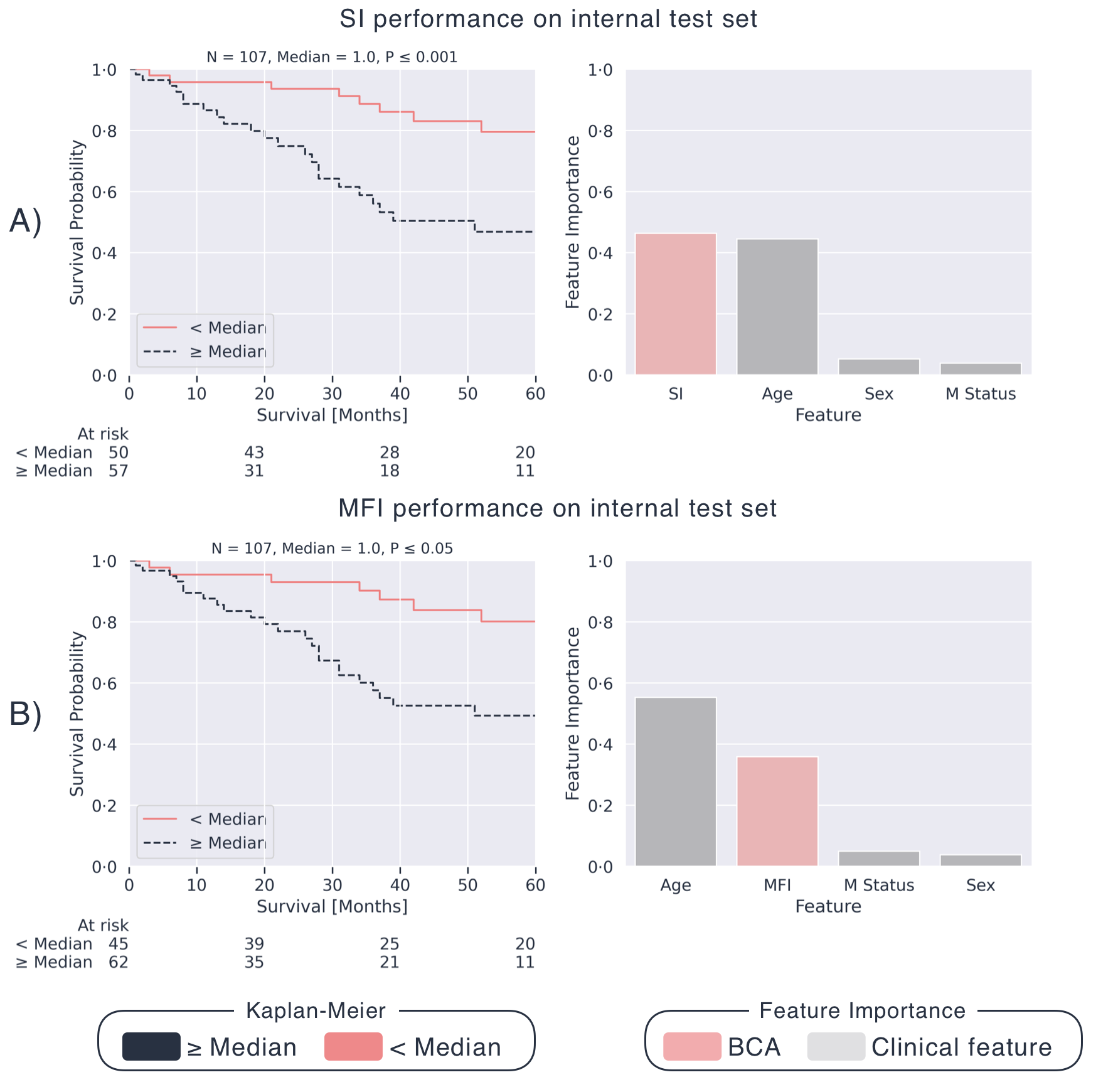

**Figure 16:** **Multivariate Machine Learning Results on Internal Thorax CT Scans:** Kaplan-Meier curves on the internal thorax test datasets for the SI (A) and MFI (B) using the predicted risk scores of the multivariable thorax-based Body Composition Analysis (BCA) models trained and tested on patients with known metastasis status (M0 or M1). Also, the averaged feature importance is shown for each model. SI: Sarcopenia Index, MFI: Myosteatosis Fat Index

# Figure 17: Restricted Mean Survival Time Differences Between Thorax-based BCA Models and the Baseline Model


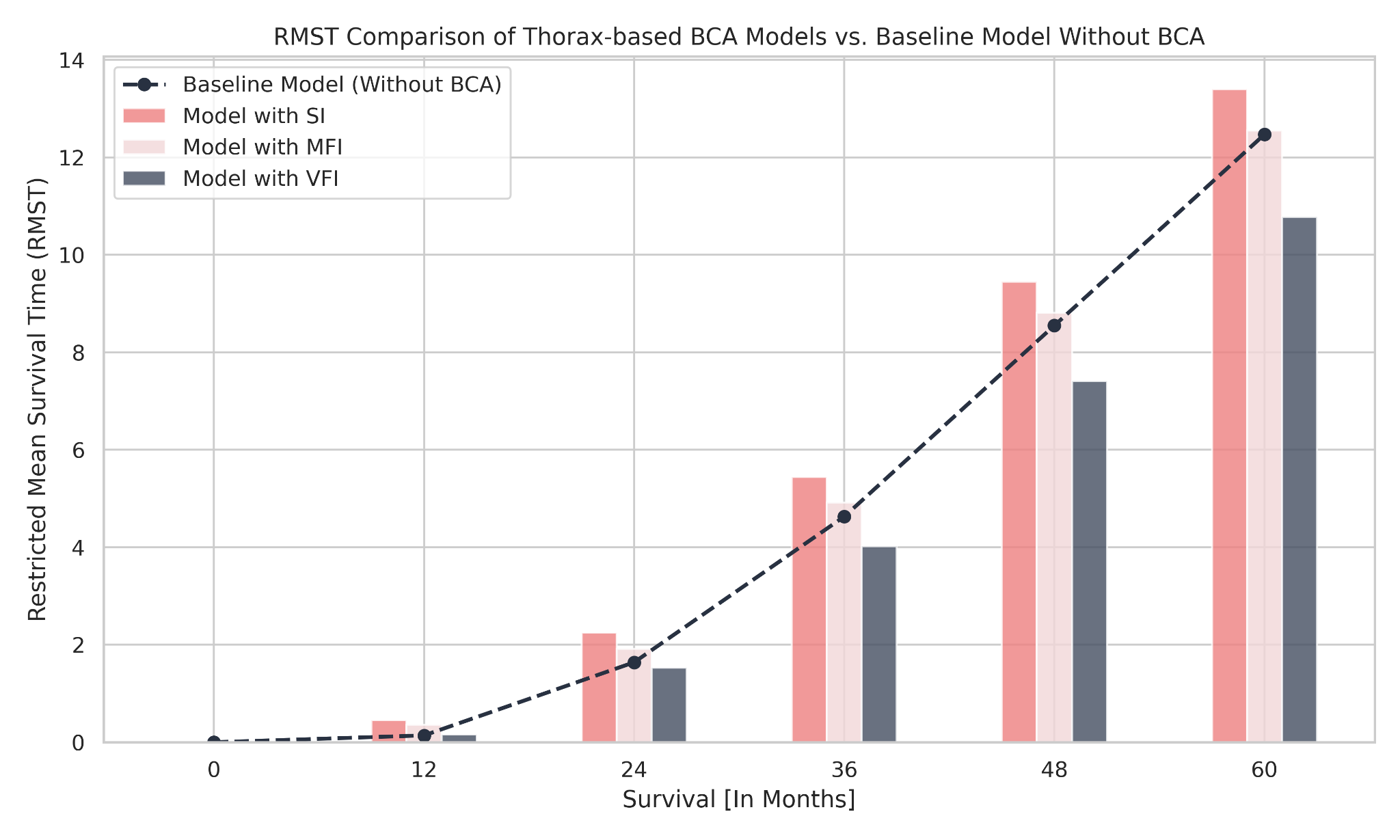

**Figure 17: Restricted Mean Survival Time Differences Between Thorax-based BCA Models and the Baseline Model:** The baseline ML model included only age at diagnosis, sex, and M status (M0 + M1), while the other models incorporated a respective BCA index in addition to these features. The RMST differences between models are presented over a 0 to 60-month range. BCA: Body Composition Analysis, ML: Machine Learning, SI: Sarcopenia Index, MFI: Myosteatosis Fat Index, VFI: Visceral Fat Index, RMST: Restricted Mean Survival Time

# Figure 18: Spline Regression for Sarcopenia Index, Myosteatotis Fat Index, and Visceral Fat Index


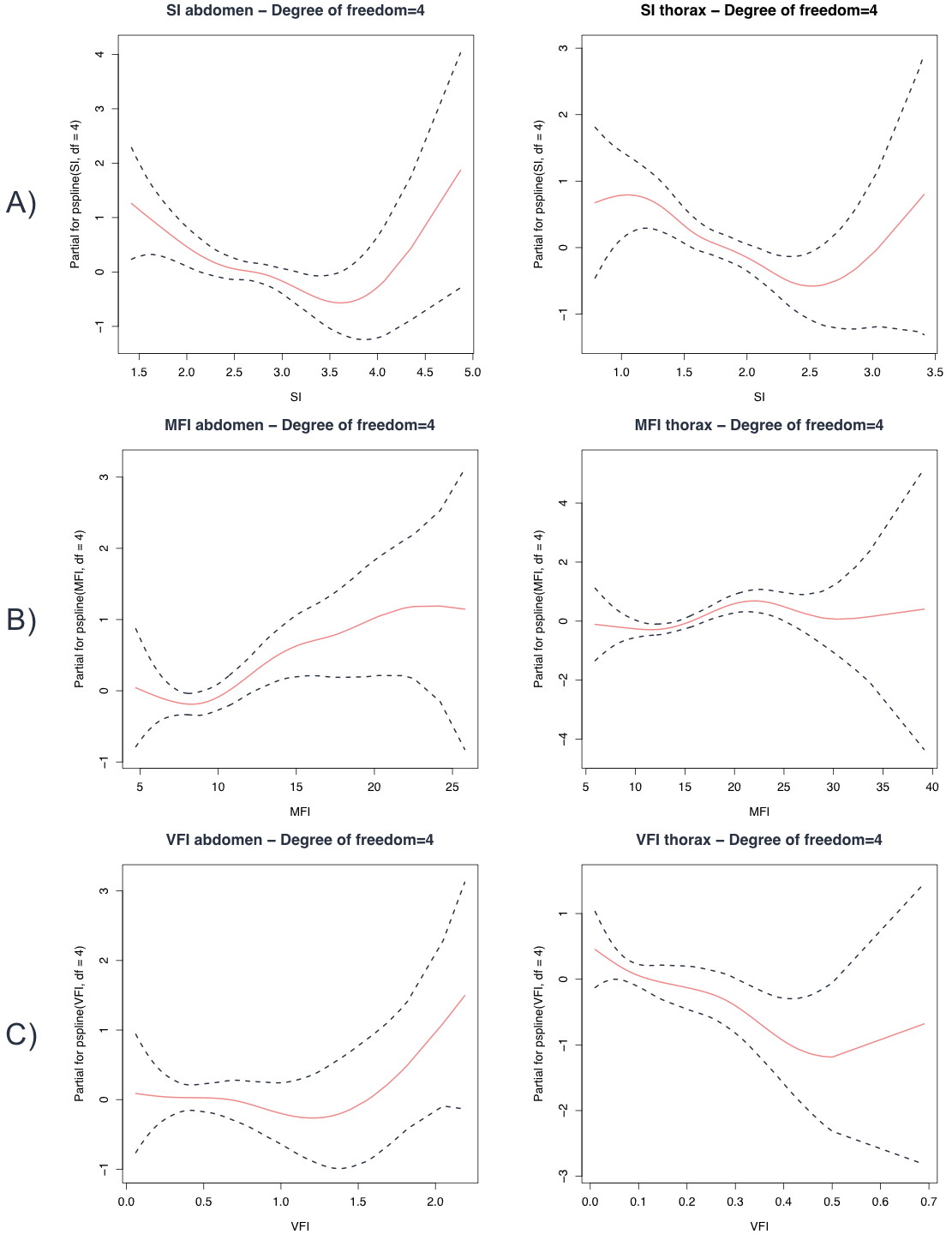

**Figure 18: Spline Regression for Sarcopenia Index, Myosteatotis Fat Index, and Visceral Fat Index:** Spline plot showing the effect of the SI (A), MFI (B), and VFI (C) on hazard ratios over their range with 4 degrees of freedom in a spline regression model. The line represents the estimated effect of the marker, while the dashed area indicates the 95% confidence interval. This analysis explores how variations in index values influence the hazard ratio and provides insight into potential non-linear relationships between the indices and the outcome. SI: Sarcopenia Index, MFI: Myosteatosis Fat Index, VFI: Visceral Fat Index

# Figure 19: Multivariate Machine Learning Results for Abdomen CT Scans in Patients With No Distant Metastases


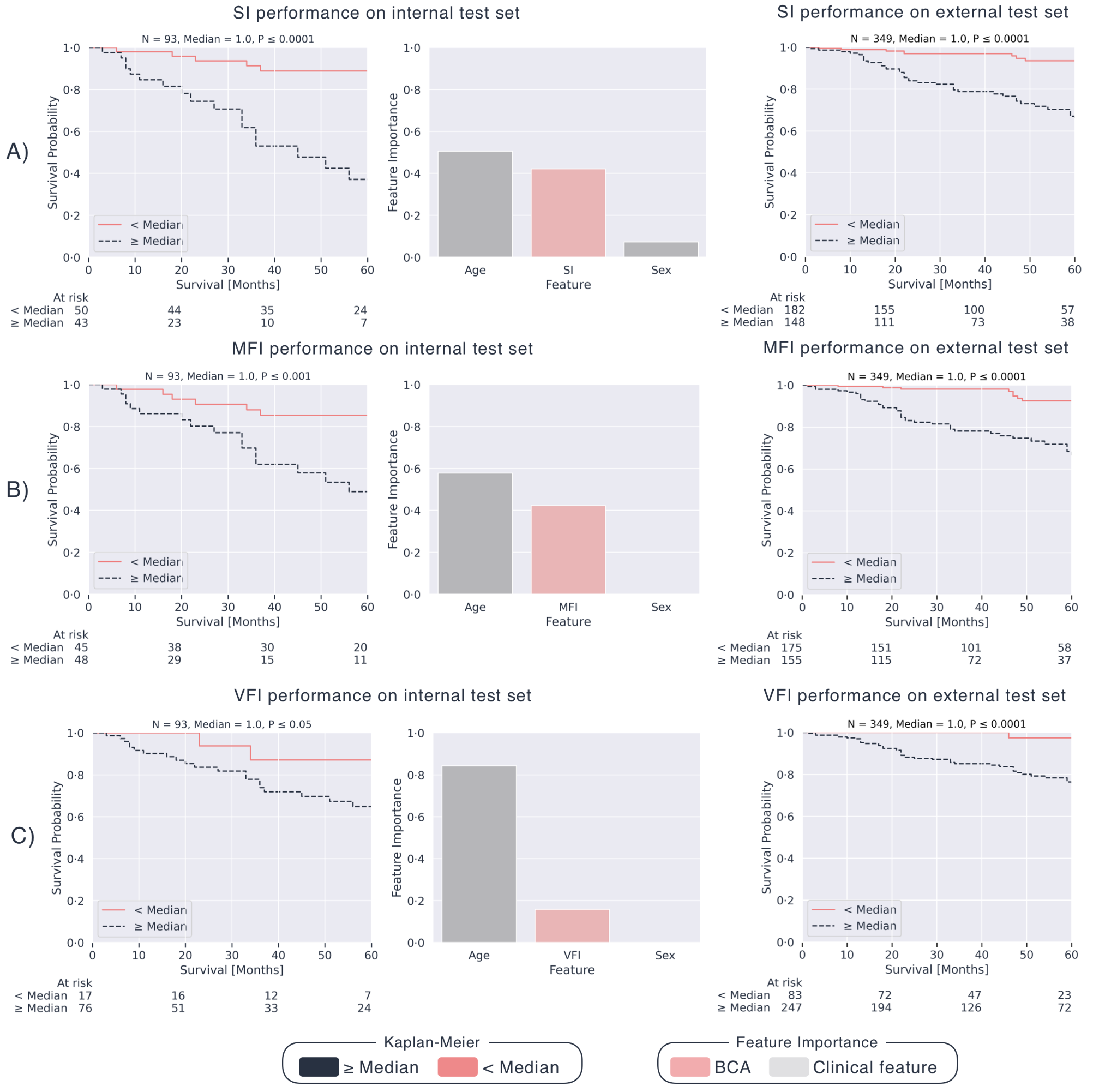

**Figure 19: Multivariate Machine Learning Results for Abdomen CT scans in Patients With No Distant Metastases:** Kaplan-Meier curves on the internal (left) and external (right) abdomen CTs for the SI (A), MFI (B), and VFI (C) using the predicted risk scores of the multivariable abdomen-based Body Composition Analysis (BCA) models trained and tested on patients with no distant metastases (M0). Also, the averaged feature importance is shown for each model. SI: Sarcopenia Index, MFI: Myosteatosis Fat Index, VFI: Visceral Fat Index

# Figure 20: Multivariate Machine Learning Results for Thorax CT Scans in Patients With No Distant Metastases


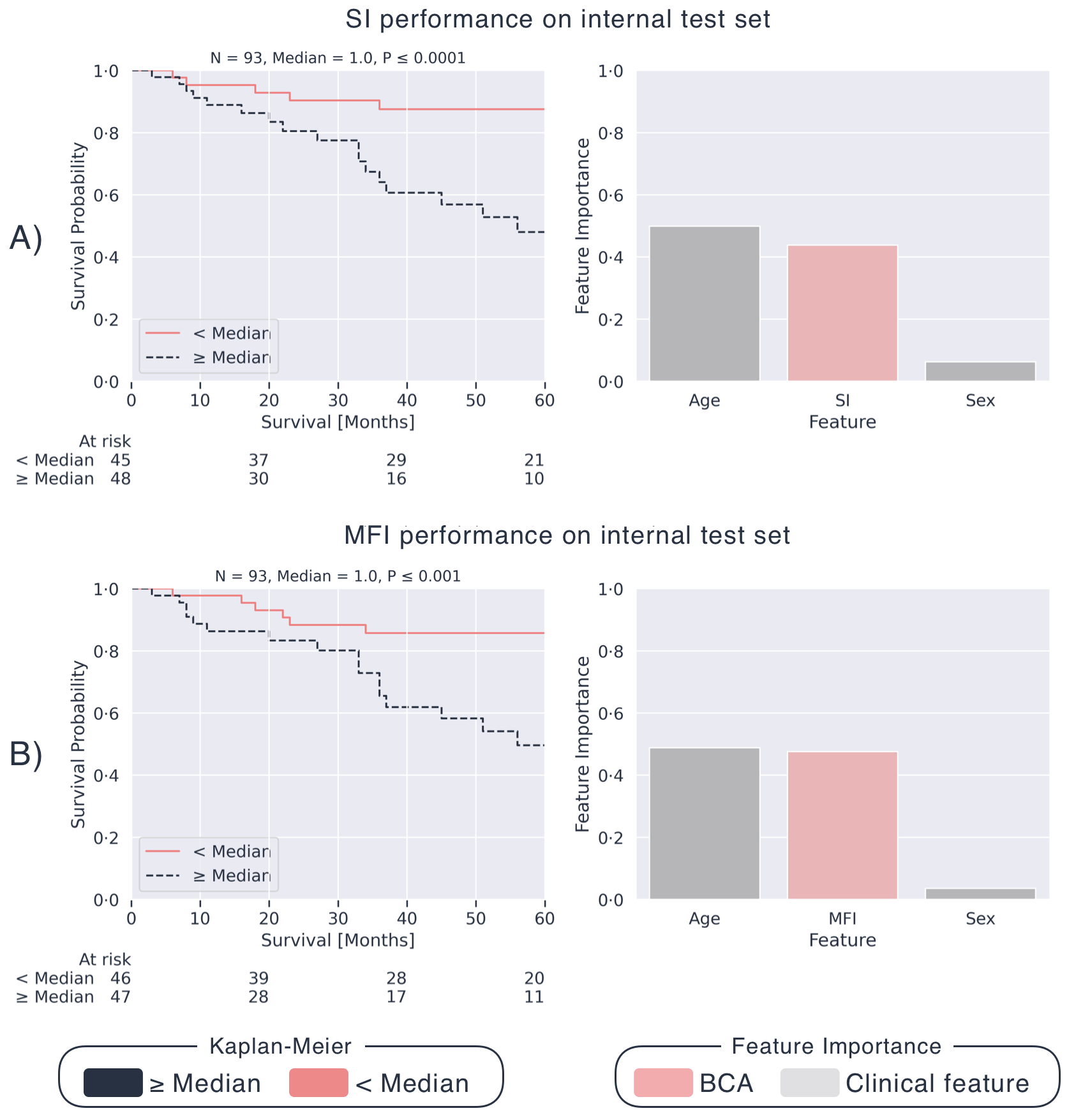

**Figure 20: Multivariate Machine Learning Results for Thorax CT Scans in Patients With No Distant Metastases:** Kaplan-Meier curves on the internal (left) and external (right) abdomen CTs for the SI (A) and MFI (B) using the predicted risk scores of the multivariable thorax-based Body Composition Analysis (BCA) models trained and tested on patients with no distant metastases (M0). Also, the averaged feature importance is shown for each model. SI: Sarcopenia Index, MFI: Myosteatosis Fat Index
